# Supplementary material for: A Phase I Dose Escalation and Expansion Study of Epidiolex (Cannabidiol) in Patients with Biochemically Recurrent Prostate Cancer
Source: Cancers (Basel). 2023 Apr 27;15(9):2505. doi: 10.3390/cancers15092505 (PMC10177512; doi:10.3390/cancers15092505)
Supplement: Supplementary file 1 [file cancers-15-02505-s001.zip › cancers-2270512-supplementary figures.pdf]

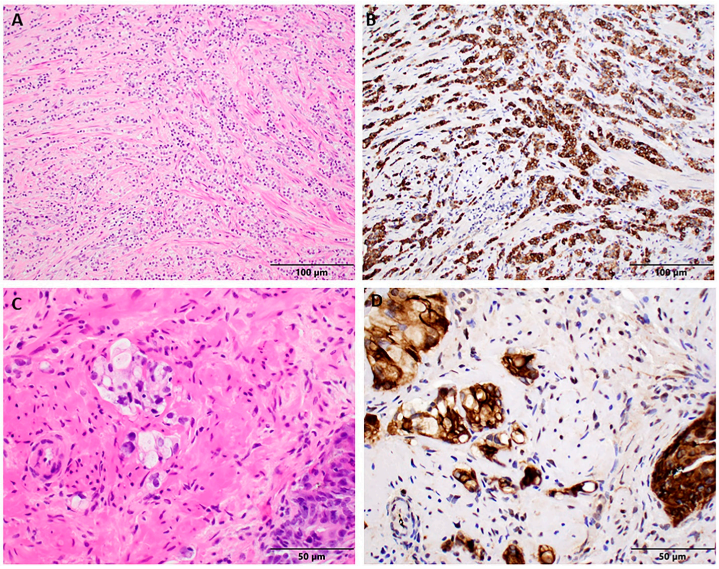

Supplement Figure S1. CB1 High Expression. A. H&E staining of high-grade prostatic adenocarcinoma composed of predominantly infiltrating poorly formed glands and single individual cells (20x magnification). **B**. Corresponding immunohistochemical staining (IHC) for CB1 expression in the same tumor showing strong and diffuse cytoplasmic and membranous expression in this field (anti-CB1 stain, 20x magnification). **C**. High-magnification of a high-grade prostatic adenocarcinoma composed of poorly formed glands (H&E stain, 40x magnification). **D**. High-magnification of corresponding IHC staining of CB1 I expression in the same tumor showing strong and diffuse cytoplasmic and membranous expression (anti-CB1 stain, 40x magnification).

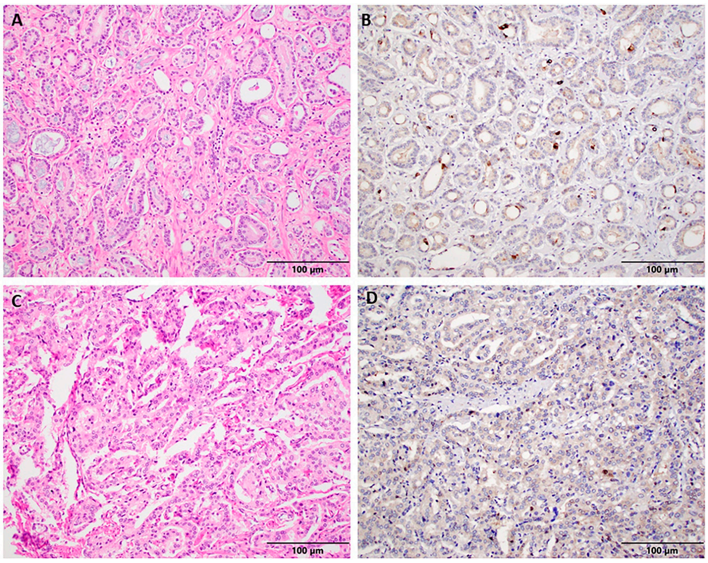

Supplement Figure S2. CB1 low expression A. H&E staining of low-grade prostatic adenocarcinoma comprised of well-formed, clearly delineated glands with scattered intraluminal mucin (20x magnification). **B**. Corresponding CB1 immunohistochemical staining (IHC) in the same tumor showing focal, scattered cytoplasmic and membranous expression (anti-CB1 stain, 20x magnification). **C**. H&E staining of intermediate-grade prostatic adenocarcinoma showing, in this field, predominantly a dense cribriform type architecture admixed with scattered poorly formed glands (20x magnification). **D**. Corresponding CB1 IHC expression in the same tumor showing only faint, rare cytoplasmic expression (anti-CB1 stain, 20x magnification).

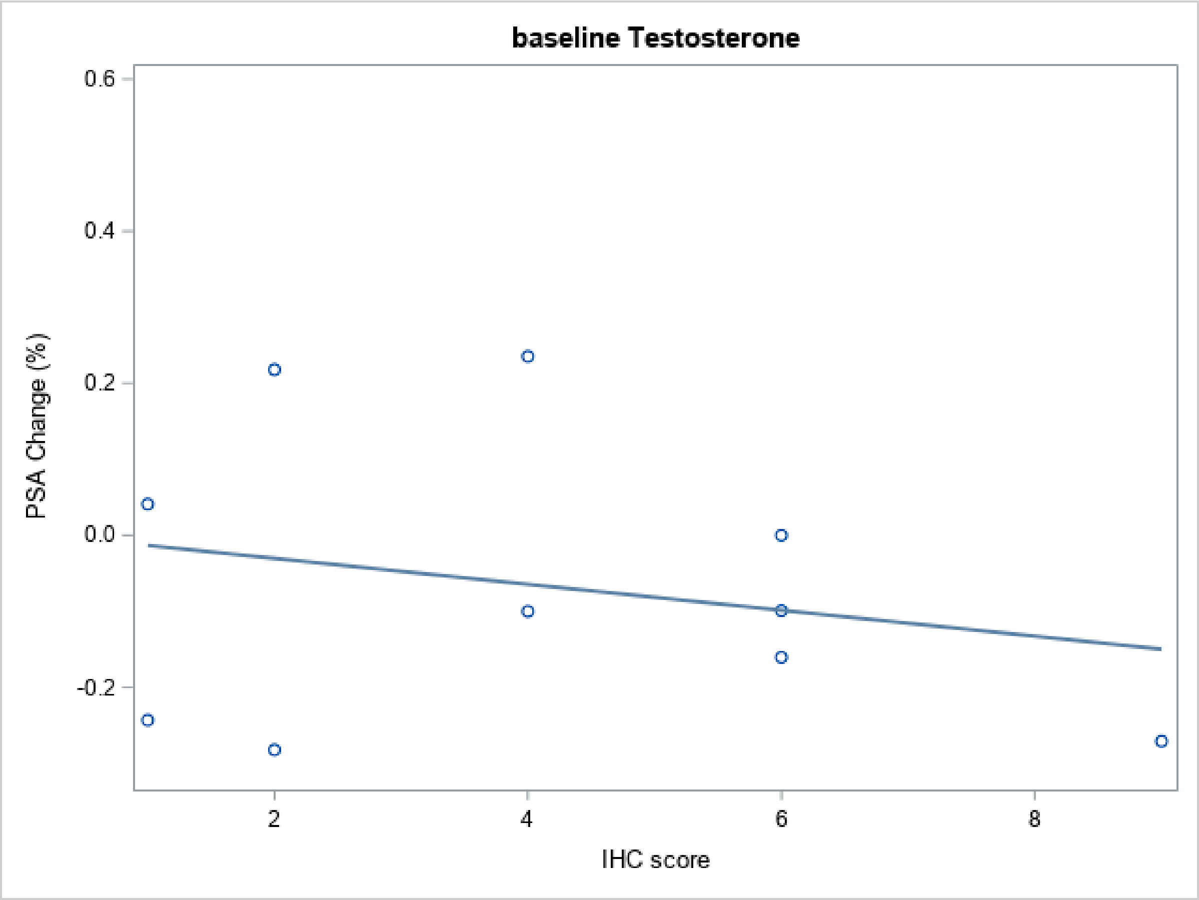

Supplement Figure S3. Correlation between CB1 IHC expression levels and PSA changes.
PSA, prostate specific antigen, IHC, immunohistochemical, CB1, cannabinoid receptor 1
