# Supplementary material for: A Phase I Dose Escalation and Expansion Study of Epidiolex (Cannabidiol) in Patients with Biochemically Recurrent Prostate Cancer
Source: Cancers (Basel). 2023 Apr 27;15(9):2505. doi: 10.3390/cancers15092505 (PMC10177512; doi:10.3390/cancers15092505)
Supplement: Supplementary file 1 [file cancers-15-02505-s001.zip › cancers-2270512-supplementary file S1.pdf]

SUMMARY OF CHANGES for AMENDMENT 2

DATE: 9/15/2020

All pages: new version date and version updates in header (changed to Amendment 2) / September 15th).
Cover Page: updates to Protocol Version and Version Date.
Table of Contents: updates to page numbers.

3.1.2, Eligibility criterion: clarified the PSA level required for biochemical recurrence after XRT to localized oligo-met.

3.1.5, Eligibility criterion: added parameters for requisite levels of total bilirubin and AST/ALT at baseline

6.1 & 11.0 Study Calendar: clarified that the window for completion of requisite baseline scans is within 12 weeks of treatment initiation (not 4 weeks).

MCC Protocol #: MCC-19-GU-74
ClinicalTrials.gov Identifier: NCT04428203

A Phase I/Ib Study on the Safety of Epidiolex in Patients with Prostate Cancer with Rising PSA after Localized Therapy with either Surgery or Radiation

Short Title: CBD in Patients with Rising PSA

PROTOCOL FACE PAGE FOR
MCC INTERVENTIONAL THERAPEUTIC PROTOCOL

ROLE	***NAME***	***DEPARTMENT/Division***
Principal Investigator	Zin W. Myint, MD	Internal Medicine/Medical Oncology
Co-Investigators	Peng Wang, MD PhD	Internal Medicine/Medical Oncology
	Andrew James, MD	Surgery/Urology
	Patrick Hensley	Surgery/Urology
	Stephen Strup, MD	Surgery/Urology
	William St. Clair, MD	Radiation Medicine
	C. Scott Ellis, PharmD	Clinical Pharmacist
	Danielle Otto, PharmD	Clinical Pharmacist
	Jill Kolesar, PharmD	Pharmacology, Precision Medicine
Collaborators	Donglin Yan, PhD	Biostatistics
	Zhonglin Hao, MD	Internal Medicine/Medical Oncology<br>Medical Director, MCC Clinical Research Organization
	Jennifer Land	Clinical Data Manager
	Derek Allison, MD	Pathology/GU pathologist
Senior Mentor	Robert S. DiPaola	Dean, College of Medicine
Investigator-Initiated Trials Office	Leigh Anne Faul, PhD	Director, IITs Office
	April Bramel	IITs Senior Project Manager

Investigational Agent: Epidiolex (Cannabidiol, FDA-approved, abbreviated as “CBD”)

Funding Source: College of Medicine / Markey Cancer Center

FDA IND Status: Study Exempt from IND Requirements per 21 CFR 312.2(b).

Protocol Type / Version Date: Amendment Version 2 / 15 SEPTEMBER 2020

Protocol Development History – Original Version to Current Version, w/ major Summary of Changes noted	
Original Protocol, 20DEC2019	PRMC full review.<br>*Resolution*: Changes required.
10JAN2020	FDA IND exemption granted, 147310.
Revision, 1<br>24JAN2020	PRMC review of revised protocol.<br>*Resolution*: Changes required.
Revision, 2<br>17FEB2020	PRMC review of the revised protocol.<br>*Resolution*: Changes required.
Revision, 3<br>10MARCH2020	Protocol revised to address PRMC feedback.
	3/16/2020 PRMC review of this revised protocol.<br>*Resolution*: PRMC full approval.
	4/23/2020 Approval from UK CRSO Medicare coverage analysis.
5/6/2020	IRB initial full review.<br>*Resolution*: IRB approval. 56982
Revision, 4<br>17JUNE2020	Protocol revised to incorporate minor edits revealed in initial mock-up of study forms build and coverage analysis. Added the NCT # from clinicaltrials.gov. Modified OnCore trial ID number to “MCC-19-GU-74” as the MCC CRO will be running the trial per email 6/10/2020. Study Calendar and for dose modifications.
	6/24/2020, PRMC Approval of Revision, protocol version 4 dated 17JUNE2020.
	6/29/2020, IRB Approval of Revision, protocol version 4 dated 17JUNE2020.
	7/24/2020: submission of patient diary to track medication compliance to IRB.<br>*Resolution*: IRB approval, 7/28/2020
**7/28/2020**	**Open to Accrual**
Amendment, v1<br>31JULY2020	Clarified language to include biochemical recurrence after primary radical prostatectomy.<br>Clarified language regarding CYP- substrates medications (section 6.5 and Appendix C).<br>Added notation on Study Calendar re: a required Day 2 phone follow-up.<br>Added Appendix F, patient diary (medication log, pre-approved by IRB 7/28/2020).
	8/12/2020, PRMC Approval
	8/17/2020, IRB Approval
Amendment, v2<br>15SEPT2020	Clarified language that the window for baseline scans is 12 weeks (not 4 weeks).<br>Added parameter to requisite baseline levels of bilirubin and AST/ALT.<br>Clarified PSA level for biochemical recurrence after radiation therapy to oligo-met.
	Date is pending, PRMC Approval
	Date is pending, IRB Approval
Amendment, v3<br>*Add date*	*Placeholder for a future amendment*
	Date is pending, PRMC Approval
	Date is pending, IRB Approval

PROTOCOL ABBREVIATIONS	
ADT	Androgen deprivation therapy
AED	Antiepileptic drug
BCR	Biochemical recurrence
CB1	Cannabinoid receptor 1
CB2	Cannabinoid receptor 2
CBD	Cannabidiol (Epidiolex)
DLT	Dose-limiting toxicity
FDA	U.S. Food and Drug Administration
IHC	Immunohistochemistry
IND	Investigational New Drug
mg	Milligrams
MTD	Maximum tolerated dose
PSA	Prostate-specific antigen
RP2D	Recommended Phase 2 Dose
THC	Delta-9-tetrahydrocannabinol

SCHEMA

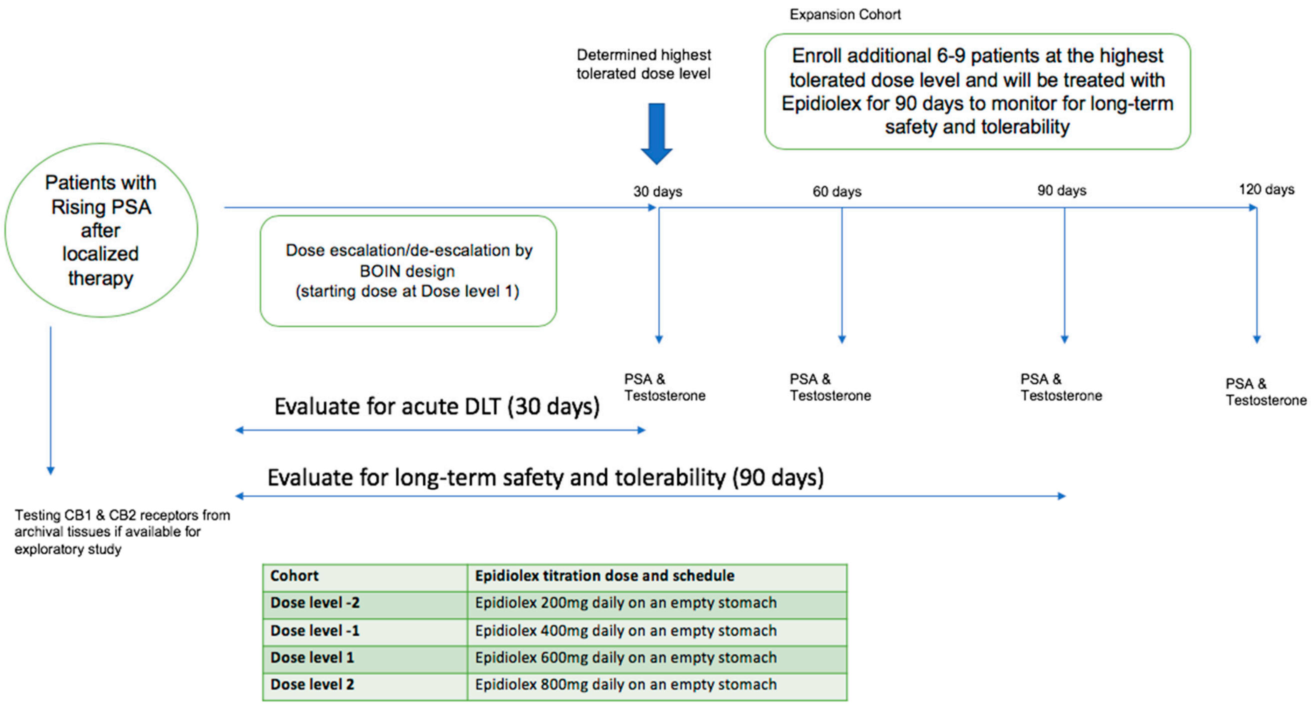

In this phase I trial of Epidiolex for biochemically recurrent prostate cancer, predefined dose levels are as follows:

Table S1. Predefined dose levels.

**Cohort**	**Epidiolex titration dose and schedule**
Dose level -2	Epidiolex 200mg daily on an empty stomach
Dose level -1	Epidiolex 400mg daily on an empty stomach
Dose level 1	Epidiolex 600mg daily on an empty stomach
Dose level 2	Epidiolex 800mg daily on an empty stomach

TABLE OF CONTENTS

1. OBJECTIVES 7
  1.1 Primary Objective 7
  1.2 Secondary Objectives....7
  1.3 Exploratory Objective 7
2. BACKGROUND....7
  2.1 Biochemically Recurrent Prostate Cancer 7
  2.2 Epidiolex 8
  2.3 Epidiolex and Cancer....10
  2.4 Study Rationale....10
  2.5 Correlative Studies Background 10
3. PATIENT ELIGIBILITY....11
  3.1 Inclusion Criteria 11
  3.2 Exclusion Criteria 12
  3.3 Inclusion of Women and Minorities 12
4. INVESTIGATOR REQUIREMENTS AND REGISTRATION PROCEDURES....13
  4.1 Protocol Review and Monitoring Committee and Institutional Review Board....13
  4.2 Investigator and Research Associate Registration with MCC....13
  4.3 Enrollment Overview....13
  4.4 Informed Consent....14
  4.5 Patient Registration....14
  4.6 General Guidelines....15
5. BIOMARKER, CORRELATIVE, AND SPECIAL STUDIES....15
6. TREATMENT PLAN 16
  6.1 Enrollment and Screening Process....16
  6.2 Administration of Epidiolex 16
  6.3 Dose Expansion Cohorts:....17
  6.4 Compliance with Oral Epidiolex 18
  6.5 General Concomitant Medication and Supportive Care Guidelines....18
  6.6 Duration of Therapy....18
  6.7 Duration of Follow-Up 19
  6.8 10-day Taper Dose Formula 19
7. DOSE MODIFICATIONS and DOSE DELAYS 20
  7.1 Dose Delays/Modifications on Hepatotoxicity during Treatment....20
  7.2 Dose Delays/Modifications on Suicidal Ideation during Treatment....20
  7.3 Dose Delays/Modifications for CNS, Hem & GI toxicities 20
  7.4 Other Grade 3 or 4 Non-hematologic toxicities....21
8. PHARMACEUTICAL INFORMATION for epidiolex....22
  8.1 Epidiolex Oral Solution, Mechanism of Action, PDs and PKs 22
  8.2 Absorption, Distribution and Elimination of Epidiolex....22
  8.3 Metabolism and Excretion 22
  8.4 Specific Populations – Hepatic Impairment 23
  8.5 Special Considerations - Drug Accountability. 23
  8.6 Drug Interaction Studies, Transporters and In Vivo 23
  8.7 Supply, Storage and Handling of Epidiolex 24
9. STATISTICAL CONSIDERATIONS....25

9.1 Study Design/Endpoints....................25
9.2 Sample Size/Accrual Rate....................26
9.3 Analysis of Primary Endpoint....................26
9.4 Analysis of Secondary Endpoints....................26
9.5 Analysis of Correlative Endpoints....................26
10. ADVERSE EVENTS: LIST AND REPORTING REQUIREMENTS....................27
10.1 Adverse Event List for Epidiolex....................27
10.2 Adverse Event characteristics....................28
10.3 MCC Expedited adverse event reporting....................29
10.4 Expedited reporting to UKHC Hospital Risk Management....................30
10.5 Routine adverse event reporting....................30
10.6 Secondary Malignancy....................31
10.7 Second Malignancy....................31
11. STUDY CALENDAR....................32
12. MEASUREMENT OF EFFECT....................35
12.1 Antitumor Effect – Solid Tumors....................35
12.2 Health-related Quality of Life....................35
13. STUDY OVERSIGHT, DATA REPORTING / REGULATORY REQUIREMENTS....................36
13.1 Study Oversight....................36
13.2 Protocol Review and Monitoring Committee and Institutional Review Board....................36
13.3 Quality Assurance....................36
13.4 Data and Safety Monitoring Committee....................37
13.5 Data Reporting....................37
13.6 Data Management....................37
13.7 Compliance with Laws and Regulations....................38
14. REFERENCES....................39
APPENDIX A: PERFORMANCE STATUS CRITERIA....................42
APPENDIX B. EORTC Quality of Life Measures....................43
APPENDIX C: PATIENT DRUG INTERACTIONS....................48
APPENDIX D: IDS Drug Accountability Form....................49
APPENDIX E: Patient Instructional Handout for Epidiolex....................51
APPENDIX F: PATIENT MEDICATION DIARY....................52

1. OBJECTIVES

1.1 Primary Objective

To evaluate the acute toxicity and long-term safety and tolerability of Epidiolex (CBD) dose in patients with biochemically recurrent prostate cancer.

Acute toxicity: defined as by experiencing a dose limiting toxicity (DLT) within 30 days after initiation of study treatment.

Long-term safety and tolerability: evaluated by AEs occurred within 90 days after initiation of study treatment.

1.2 Secondary Objectives

1.2.1 To measure change in serial PSA, PSA velocity and testosterone levels from baseline throughout the treatment period as an indication of biochemical response

1.2.2 To assess health-related quality of life (EORTC QLQ-C30 and QLQ-PR25)

1.3 Exploratory Objective

1.3.1 Primary tumor from prostatectomy specimen will be assessed for CBD receptor 1 and 2 expression levels, among patients for whom archival surgical specimens are available.

2. BACKGROUND

2.1 Biochemically Recurrent Prostate Cancer

Biochemically recurrent (BCR) prostate cancer is an increasingly common disease state, with more than 25,000 cases annually [1]. Approximately 30-40% of all prostate cancer patients will develop biochemical or PSA recurrence within 10 years [2]. Whereas, high-risk localized prostate cancer patients (50-90%) have a higher rate of BCR progression [1]. In 2007, the American Urological Association defined biochemical recurrence after radical prostatectomy as an initial PSA level of ≥ 0.2 ng/ml and with a second test confirming levels of > 0.2 ng/ml without radiological or clinical progression [3]. The natural history of PSA recurrence is usually long but can be varied. In a longitudinal cohort of 379 men followed for 22 years, the median time from PSA recurrence to prostate cancer death was not reached after 16 years [4]. In spite of this, prostate cancer deaths are occasionally seen as early as 1 year after PSA recurrence. Thus, although the natural history of recurrent prostate cancer is often one of a slowly progressive disease spanning years or decades, it can also be very rapid in a subset of patients. Moreover, 90% of all recurrences after radical prostatectomy were found within 5 years of prostatectomy as per Duke Prostate Cancer Database [5]. Patients who had early (≤5 years) PSA recurrence have greater risk for cancer-death compared to those that recur >5 years post radical prostatectomy [5]. It has been shown that rapid prostate specific antigen doubling time (PSADT) is closely linked with risk for prostate cancer death and overall survival [4, 6]. Among men with a PSADT > 3 months after either RP or RT, PSADT as a continuous variable was significantly associated with prostate cancer death [6]. It has become common in clinical practice to make treatment decisions for men with PSA recurrence based on small changes in PSA level, PSADT, Gleason score and time to PSA recurrence [4, 8-10]. The clinical entity of recurrent prostate cancer is common, affecting many current era patients and the subject of many encounters by urologists, radiation oncologists, and medical oncologists.

Isolated biochemical recurrence of prostate cancer is challenging to treat because of the absence of radiographic disease to monitor response. There is no clear consensus on when to begin androgen deprivation therapy (ADT), and the optimal duration of ADT in men with BCR. ASCO updated consensus panel recommendations in 2007, the authors refrained from strongly recommending early ADT initiation [11]. The Veterans Administration Cooperative Urological Research Group found no difference in overall survival when they compared early with deferred hormonal therapy [12]. Moul et al. reviewed data from 1,352 men with postsurgical PSA recurrence and found that, early ADT had no effect on time to metastasis [13]. However, among those with high-risk disease, (pathologic Gleason ≥8 or PSADT <12 months, early ADT (i.e., starting when the PSA was <5 or < 10 ng/ml) was associated with a 50% reduction in the risk of metastasis after a median follow-up of 3.7 years after PSA recurrence [13]. Unfortunately, follow-up was too short to assess the association between timing of ADT and prostate cancer mortality. Garcia-Albenix et al. analyzed 2,096 men experiencing biochemical recurrence after initial treatment from the CaPSURE registry (Cancer of the Prostate Strategic Urologic Research Endeavour) [14]. The effect of immediate ADT initiation (within 3 months of PSA relapse) on overall survival and prostate-cancer specific survival was evaluated, in comparison to deferred ADT (initiated at the development of metastases, symptoms or a short PSA doubling time). The adjusted mortality hazard ratio for immediate ADT versus deferred ADT was 0.91 (95% CI, 0.52-1.60), demonstrating no significant advantage to early initiation of ADT [14]. ADT confers significant decrements to functioning and quality of life, including sleep disturbance, hot flushes, breast enlargement and tenderness, mood swing, and increased risk of cardiovascular-related death have been observed [15-16]. Thus, the treatment decision for biochemical recurrence is complicated by the need to balance the efficacy of the therapy (gains in overall and disease-specific survival) against minimization of side effects and decline in quality of life in this generally asymptomatic population.

Myriad alternative therapies for BCR have been investigated: celecoxib treatment for post-RP BCR decreased PSA velocity [17]; increased intake of vegetable proteins while decreasing animal protein and saturated fat consumption [18] or intake of a soy-based dietary supplement prolonged PSA velocity among men with BCR [19]. NCCN recommends in those population expectant management is also appropriate given the known toxicity of ADT [20]. Thus, ongoing investigation and development of non-hormonal therapies is in demand.

2.2 Epidiolex

Cannabis is derived from the *Cannabaceae* family of plants, which contain more than 400 chemical components; 80 of which are cannabinoids and 200 non-cannabinoids [21]. The major cannabinoids compounds are THC (Delta-9-tetrahydrocannabinol) and CBD (Cannabidiol). Cannabinoids have central (CB1) receptor and peripheral (CB2) receptor [22-23]. CBD, extracted from hemp, *Cannabis sativa* plant species, lacks a high concentration of THC, thus, it does not cause a psychoactive effect [21].

The cannabis plant *(Cannabis sativa L.)* produces trichomes that synthesize a large number of pharmacologically active compounds called phytocannabinoids. The most abundant of these are delta-9-tetrahydrocannabinol (THC) and cannabidiol. The amount and proportions of the various phytocannabinoids in each plant vary by strain and can be adjusted by breeding.

Epidiolex, an FDA-approved CBD agent, is formulated from extracts prepared from *Cannabis sativa L.*

plants that have a defined chemical profile and contain consistent levels of CBD as the principal phytocannabinoid. Extracts from these plants are processed to yield pure CBD (>95%) that typically contains less than 0.5% THC. The pure CBD extract is subsequently dissolved in excipients with added sweetener and flavoring. **In June 2018, the FDA approved Epidiolex in the treatment of seizure disorder associated with Lennox-Gastaut syndrome or Dravet syndrome in patients 2 years of age and older with the maximum dose of 600-800mg per day.**

The pharmacological effects of phytocannabinoids are mediated primarily via their interaction with the endocannabinoid system, which consists of cannabinoid receptors, endogenous ligands (endocannabinoids) and enzymes for endocannabinoid synthesis and degradation. To date, two G-protein-coupled receptors for cannabinoids have been identified, designated as cannabinoid CB1 and CB2. CBD does not bind to either of these receptors with any great affinity but does modulate the metabolizing enzymes of the endocannabinoid system. CBD also affects conduction of ion channels and acts on other G-protein-coupled receptors such as TRPV1 [24] and GPR55 [25]. Importantly, CBD generally lacks detectable psychoactivity, as found with THC. Additionally, CBD has demonstrated anticonvulsant, antipsychotic, anxiolytic, neuroprotective, antioxidant and anti-inflammatory activity [26]. Very little data concerning AEs of CBD in humans exists to date. However, doses of up to 1500 mg CBD per day are reported to be well tolerated in humans [27].

Epidiolex and Lennox–Gastaut Syndrome (LGS)

The effectiveness of EPIDIOLEX for the treatment of seizures associated with LGS was established in two randomized, double-blind, placebo-controlled trials in patients aged 2 to 55 years. Study 1 (N=171) compared a dose of EPIDIOLEX 20 mg/kg/day with placebo [28]. Study 2 (N=225) compared a 10 mg/kg/day dose and a 20 mg/kg/day dose of EPIDIOLEX with placebo [29]. In both studies, patients had a diagnosis of LGS and were inadequately controlled on at least one antiepileptic drug (AED), with or without vagal nerve stimulation and/or ketogenic diet. Both trials had a 4-week baseline period, during which patients were required to have a minimum of 8 drop seizures (≥2 drop seizures per week). The baseline period was followed by a 2-week titration period and a 12-week maintenance period. In Study 1, 94% of patients were taking at least two concomitant AEDs. The most frequently used concomitant AEDs (>25%) in Study 1 were clobazam (49%), valproate (40%), lamotrigine (37%), levetiracetam (34%), and rufinamide (27%). In Study 2, 94% of patients were taking at least two concomitant AEDs. The most frequently used concomitant AEDs (>25%) in Study 2, were clobazam (49%), valproate (38%), levetiracetam (31%), lamotrigine (30%), and rufinamide (29%).

Epidiolex and Dravet Syndrome

McCoy et al. studied dosing and tolerability of CBD in pediatric patients with Dravet syndrome (also known as Severe Myoclonic Epilepsy of Infancy). In that study, the starting dose was 2 mg/kg/day CBD divided twice daily with weekly titration by 2 mg/kg/day every 7 days up to a maximum dose of 16 mg/kg/day [14]. Nineteen out of 20 pediatric patients completed the 20-week intervention and the mean dose achieved was 13.3 mg/kg/day of CBD. The most common adverse events included somnolence, anorexia, diarrhea and some abnormal liver transaminases [30].

2.3 Epidiolex and Cancer

Cannabinoids have been widely used in medicines for centuries to control pain, nausea or vomiting, and to stimulate appetite, especially in cancer patients [31-34]. In 1974, the discovery of cannabinoids' antagonist effect in the male reproductive system has attracted much attention in prostate cancer research [35]. Kolodny et al. examined testosterone and chronic intensive marijuana use (defined as four or more days a week for a minimum of six months) [35]. Plasma testosterone level was significantly reduced by almost 50% among intensive-use marijuana participants compared to never-users [35].

Interestingly, Sarfaraz et al. demonstrated that both CB1 and CB2 receptors were highly expressed in cultured prostate cancer cells compared to normal prostate cell lines. Two cell lines (PrEC & LNCaP) were treated with WIN-55 212-2, a potent cannabinoid receptor agonist, for 24- and 48-hours and evaluated the cell viability [36]. It demonstrated significant decreases in LNCaP cells viability at 24- and 48-hours in a dose-dependent manner, but not in the PrEC cells. The same cell lines were pretreated with SR141716 (CB1 antagonist) or SR144528 (CB2 antagonist) and subsequently, administered WIN-55 212-2. This pretreatment did not affect cell viability, suggesting that both CB1 and CB2 receptors may be involved in the growth inhibition. Results also showed a significant decrease in PSA protein expression by the dose-dependent effect of WIN-55 212-2 on LNCaP cells [36]. Increased apoptosis of prostate cancer cells when treated with cannabinoid agonists is potentially via a mechanism of cell cycle arrest G0/G1 phase by downregulation of cyclin D, cyclin-dependent kinases, and induction of p53 [37].

The effect of CBD and BDS (botanical drug substance) were studied in xenograft tumors from LNCaP and DU-145 cells in athymic mice. Those mice were divided into six different groups and treated with CBD BDS, bicalutamide, and also co-administered with docetaxel or bicalutamide at different doses. [22] It showed that CBD BDS significantly inhibited xenograft growth and combined CBD-BDS with bicalutamide significantly prolonged survival as compared with bicalutamide or CBD-BDS alone after 47 days of treatment [38].

2.4 Study Rationale

Until now, clinicians have been challenged to improve the treatment of biochemically recurrent (BCR) prostate cancer in which prostatic specific antigen (PSA) rises without radiological or clinical progression years after localized treatment (radical prostatectomy or radiation therapy) with or without hormonal treatment. Approximately 50-90% of men with high-risk prostate cancer will experience a BCR. [1] Based on the abovementioned preclinical observations of CBD's effect on prostate cancer and its safety data in two non-cancer populations, we propose to conduct a phase I study of CBD in men with biochemically recurrent prostate cancer, using the study schema shown above.

2.5 Correlative Studies Background

Cannabinoids have central (CB1) receptor and peripheral (CB2) receptor. CB1 is mainly active in the brain, lungs, and reproductive organs, whereas, CB2 is in the immune system and the bones. THC primarily works on CB1 receptors and CBD on CB2 receptors [39]. Sarfaraz et al. demonstrated that both CB1 and CB2 receptors were highly expressed in cultured prostate cancer cells compared to normal prostate cell lines [36]. We will examine CB1 and CB2 expression in available prostate cancer archival tissue in order to test the viability as a potential biomarker.

3. PATIENT ELIGIBILITY

3.1 Inclusion Criteria

3.1.1 Completion of localized therapy (prostatectomy or radiotherapy) for prostate adenocarcinoma (either histologically or cytologically confirmed)

3.1.2 Biochemical (PSA) recurrence, defined as:
- PSA of ≥0.2 ng/ml that has increased above nadir following radical prostatectomy;

<u>or</u>

- PSA increase of 2.0 ng/ml above post-therapy nadir after primary radiotherapy.

or

- PSA ≥ 0.2 ng/ml after primary radical prostatectomy followed by salvage radiotherapy and/or radiation therapy to the localized oligo-met (either bone or lymph nodes).

NOTE: PSA measured at two consecutive timepoints (separated by 4 or more weeks) is required in order to demonstrate the requisite increase in PSA

3.1.3 Age ≥18 years.

3.1.4 ECOG performance status ≤2 (see Appendix A).

3.1.5 Adequate organ and marrow function at baseline (pre-study) as defined below:

– absolute neutrophil count	≥1,500/mcL
– platelets	≥80,000/mcL
– Total Bilirubin:	≤ 1.5 x institutional upper limit of normal
– AST(SGOT)/ALT(SGPT)	≤ 3 x institutional upper limit of normal
– glomerular filtration rate (GFR)	≥30 mL/min/1.73 m^2 using the Cockcroft-Gault formula

3.1.6 Patients with a prior or concurrent malignancy (non-prostate) whose natural history or treatment does not have the potential to interfere with the safety or efficacy assessment of the investigational regimen as determined by the treating physician are eligible.

3.1.7 Given that worsening of an underlying state of mental depression or suicidal ideation has been reported with Epidiolex, patients should be carefully screened for depression at baseline and if there are indications or a history of depression it is strongly recommended that these patients be closely followed together with behavioral health or psychiatric medical support. Patients with an established diagnosis of depression that, in the assessment of the investigator may make the administration of Epidiolex hazardous, should not be enrolled on this protocol.

3.1.8 Ability to understand and the willingness to sign a written informed consent document.

3.2 Exclusion Criteria

3.2.1 History of hypersensitivity to Epidiolex (cannabidiol) or sesame seeds (one of the inactive ingredients in Epidiolex)

3.2.2 Any radiological evidence of metastatic disease (determined by standard of care CT scans of abdomen. pelvis, chest, whole body bone scan or Axium PET/CT scan). Questionable lesions on bone scan will be confirmed by standard of care methods such as plain X-rays or Axium PET/CT scan, if not previously performed.

3.2.3 Receipt of prior cytotoxic chemotherapy for recurrent prostate cancer

3.2.4 Use of androgen deprivation therapy (for example, bicalutamide, flutamide, niluamide, or leuprolide acetate) concurrently or within the previous 3 months.

3.2.5 Uncontrolled intercurrent illness such as active infections. Other illnesses will be evaluated and eligibility status determined at the discretion of the treating physician and the investigator.

3.2.6 Psychiatric illness/social situations that would limit compliance with study requirements.

3.2.7 Concomitant use of Valproate or Clobazam.

3.2.8 Concurrent use of over-the-counter CBD oil, Marinol or marijuana is not permitted. Patients with a history of current over-the-counter CBD oil, Marinol or marijuana use for any reason are **eligible <u>only</u> if** they do the following:

- complete a one-week washout period prior to study initiation
- refrain from non-study related CBD oil, Marinol or marijuana use while on-study

3.2.9 Treatment with sensitive substrates of CYP2C19 inhibitors should not be taken within 14 days prior to first dose of study treatment and for the duration of study.

3.3 Inclusion of Women and Minorities

Prostate Cancer occurs exclusively in men, so women are excluded from this study.

4. INVESTIGATOR REQUIREMENTS AND REGISTRATION PROCEDURES

4.1 Protocol Review and Monitoring Committee and Institutional Review Board

Before implementing this study, the protocol must be reviewed by the Markey Cancer Center's Protocol Review and Monitoring Committee (PRMC). Additionally, the protocol, the proposed informed consent form and other information to subjects, must be reviewed by the University of Kentucky Institutional Review Board (IRB). A signed and dated UK IRB initial review approval memo must be maintained in the Markey Cancer Center Clinical Research Office (MCC CRO) regulatory binder. Any amendments to the protocol, other than administrative ones, must be reviewed and approved by the PRMC, and UK IRB.

4.2 Investigator and Research Associate Registration with MCC

All investigators must be qualified by education, training and experience to assume responsibility for the proper conduct of human subject research. Investigators are responsible for being able to provide evidence of such qualifications through up-to-date curriculum vitae and/or other relevant documentation and training per institutional, state and federal guidelines. All investigators conducting MCC trials will register with the MCC Clinical Research Office and complete all requisite training and registrations per MCC SOPs. Contact the MCC-CRO Regulatory unit at mccreg@uky.edu if you need assistance in completing your NCI registration/renewal.

4.2.1 Delegation of Tasks Log (DTL)

All MCC studies require a Delegation Task Log, which is maintained by the MCC Regulatory Unit of the Clinical Research Office. In order to be added to the DTL for this study, each staff member must have protocol specific training and review of the final protocol. Site initiation visit training will be set up by the MCC Regulatory and Quality Assurance Program members. The DTL log will identify the protocol version on which each staff member was trained when being added to a study.

4.3 Enrollment Overview

Eligible patients will be identified by the principal investigator and co-investigators of this study. Potentially eligible patients will be screened in the University of Kentucky Markey Cancer Center clinics by the investigators and study personnel with oversight by the Principal Investigator (PI). Upon obtaining proper consent, potentially eligible patients will be registered in OnCore database by the study staff. During the screening and enrollment process, registering individuals (study staff and PI) will be required to complete a trial-specific Eligibility Checklist for each patient. The PI or treating physician signing the Eligibility Checklist is confirming whether or not the patient is eligible to enroll in the trial. Upon confirmation of eligibility, the patient will be enrolled into the trial (i.e., on-study date is entered in OnCore).

4.4 Informed Consent

The goal of the informed consent process is to provide sufficient information so that potential participants can make informed choices about whether to begin or continue participation in clinical research. The process involves a dynamic and continuing exchange of information between the research team and the participant throughout the research experience. It includes discussion of the study's purpose, research procedures, risks and potential benefits, and the voluntary nature of participation.

The informed consent document provides a summary of the clinical study and the individual's rights as a research participant. The document acts as a starting point for the necessary exchange of information between the investigator and potential research participant. Also, research participants and their families may use the consent document as an information resource and reference throughout participation in the trial. The informed consent document is often considered the foundation of the informed consent process; it does not, however, represent the entirety of the process. Nor is the informed consent document a risk-management tool for the investigator and/or institution.

The investigator must explain to each subject (or legally authorized representative) the nature of the study, its purpose, the procedures involved, the expected duration, the potential risks and benefits involved, any discomfort it may entail. Each subject must be informed that participation in the study is voluntary and that he may withdraw from the study at any time and that withdrawal of consent will not affect his subsequent medical treatment or relationship with the treating physician.

The informed consent shall be provided as a standard written statement, written in non-technical language. The subject should read and consider the statement before signing and dating it, and should be given a copy of the signed document. If the subject cannot read or sign the documents, oral presentation may be made or signature given by the subject's legally appointed representative, if witnessed by a person not involved in the study, mentioning that the patient could not read or sign the documents. No patient can enter the study before his informed consent has been obtained. The informed consent form is considered to part of the protocol, and must be submitted by the investigator with the protocol at the time of IRB review.

4.5 Patient Registration

To enroll a patient, the following information should be reviewed by the Clinical Research Nurse (CRN) / Clinical Research Associate (CRA) with the study physician per MCC SOPs to confirm eligibility:

- Copy of required laboratory tests
- Pathology reports
- Physician dictations
- Imaging reports
- Signed patient consent form
- HIPAA authorization form
- Referring physician records as available
- Other required screening procedures when applicable
- Eligibility Checklist

Once eligibility is confirmed, the CRN/CRA will complete subject enrollment to the trial in the OnCore database. To complete the enrollment process, the CRN/CRA will complete the OnCore on-study form, which comprises the following:

- Assignment of a patient study number
- Diagnosis, Date of Diagnosis
- Enter an On-Study Date
- receive the automated dose assignment if appropriate
- send an email to the study team, if randomization or dose level assignments are required.

4.6 General Guidelines

Following registration, patients should begin protocol treatment within 21 days. Issues that would cause treatment delays should be discussed with the Principal Investigator. If a patient does not receive protocol therapy following registration, the patient's registration on the study may be canceled. The study team should be notified of cancellations as soon as possible.

5. BIOMARKER, CORRELATIVE, AND SPECIAL STUDIES

Primary tumor from prostatectomy specimen (archival tissue only, if available) will be assessed for CBD receptor 1 and 2 expression levels if the surgical specimens are available.

Specimen Collection and Handling		
Archival Tissue	• *Formalin-fixed paraffin-embedded (FFPE) tumor tissue block (preferred) from the existing biobank of Biospecimen Procurement and Translational Pathology SRF services* A copy of the corresponding anatomic pathology report must be sent with the archival tissue.<br>*If a block is not available, then submit:*<br>• *1 H&E stained slide*<br>• *30-50 unstained, uncharged, unbaked slides*	**Send Archival Tissue Specimens to:**<br>Dr. Derek Allison, Biospecimen Procurement and Translational Pathology Shared Resource Facility (BPTP SRF).
Research Labs For Correlative Studies	• *Blood specimens for PSA and total testosterone*	Research Labs are drawn by phlebotomists in clinic and sent to institutional central lab for processing and analysis

6. TREATMENT PLAN

6.1 Enrollment and Screening Process

Patients who satisfy all inclusion and none of the exclusion criteria specified in Section 3 will then be approached to participate in the study. Prior to any study-required tests, subjects must first provide written informed consent to participate in this study. All lab tests should be completed within 4 weeks prior to initiation of treatment and radiographic studies should be completed within 12 weeks prior to registration/initiation of treatment.

Within 4 weeks of enrollment, all patients will undergo a history and physical exam, ECOG performance status evaluation, complete blood count with differential and platelets, and serum chemistries (including sodium, potassium, chloride, bicarbonate, calcium, total protein, albumin, BUN, creatinine, AST, alkaline phosphatase, and total bilirubin), a PSA and a pre-screening with distress thermometer score to identify any underlying depression or suicidal ideation. Standard of care scans will be used to verify eligibility and appropriate stage of disease.

6.2 Administration of Epidiolex

Treatment with Epidiolex will be administered on an outpatient basis. Participant cohorts will be instructed to take once a day on an empty stomach (either one hour prior to meal or two hours after meal) as the plasma drug concentration can be increased up to 4-fold with high fat food. The Epidiolex dose will be given a fixed-dose to all individuals.

The dose-escalation and de-escalation will be assessed by using BOIN design. The first cohort will be enrolled in the dose level 1 (600mg once daily on an empty stomach) and will be monitored for DLT in the first 30 days. Dose levels for the subsequent cohorts will be based on the BOIN design. Patients will be treated for total 90 days with additional 10 days tapering dose and will be monitored 90 days for long-term safety and tolerability.

Reported adverse events and potential risks are described in Sections 6.5, 8.6 and 10. Appropriate dose modifications described in Sections 6.2.1, 6.5 and 7.

Any kind of androgen deprivation therapy is **NOT** allowed during the study.

Table S2. Pre-defined dose levels of Epidiolex

**Cohort**	**Epidiolex titration dose and schedule**
Dose level -2	Epidiolex 200mg oral solution once daily on an empty stomach
Dose level-1	Epidiolex 400mg oral solution once daily on an empty stomach
Dose level 1	Epidiolex 600mg oral solution once daily on an empty stomach
Dose level 2	Epidiolex 800mg oral solution once daily on an empty stomach

10-day Dose Taper when Epidiolex is completed/discontinued: see Section 6.7

Subjects will receive an instructional handout on how to take Epidiolex (Appendix E). Investigators will watch closely for suicidal ideation and worsening depression during this trial and address any

changes in an ongoing manner (Sections 6.2.1, 6.6, 7.2 and 10).

6.2.1 Definition of Dose-limiting Toxicity (DLT):

To be evaluable for DLT patients must receive at least 75% of the planned dose of Epidiolex in the first 30 days of the treatment, unless the reason they did not receive study medication was toxicity. DLT will be defined as the occurrence of any of the following events in the first 30 days of treatment in the dose escalation cohorts that is **possibly, probably or definitely** related to study drug.

Hematologic
- Febrile neutropenia (ANC <1000/ul and temperature ≥ 38.5C)
- Grade 4 neutropenia lasting > 5 days
- Grade 3 thrombocytopenia with bleeding
- Grade 4 thrombocytopenia
- Grade 4 anemia that is not explained by underlying disease

Gastrointestinal
- Grade 3 or 4 nausea, vomiting, or diarrhea that persists >72 hours despite optimal anti-emetics and anti-diarrheal treatment

Suicidal Ideation
- CTCAE v5.0 Grade 2 or greater Suicidal Ideation during the treatment period, as evaluated by the treating investigator and per Markey's guidelines for identification and triage of distress, which follows the NCCN Distress Management clinical practice guidelines use of the validated Distress Thermometer at each clinic visit.

Others
- Any Grade 3 or greater adverse effect OR any > Grade 2 not improved after stopping the drug for more than a two-week period assuming all toxicities are related to Epidiolex and occur during the first 30 days of treatment.

Toxicity is per graded by the Common Terminology Criteria for Adverse Events version 5.0, or any subsequent version released during the conduct of this study. Please refer to the following resource for further details. https://ctep.cancer.gov/protocoldevelopment/electronic_applications/docs/CTCAE_v5_Quick_Reference_5x7.pdf .

Patients requiring a delay of more than 2 weeks or more than two dose reductions should go off protocol therapy. Management and Dose Modifications associated with the above adverse events are outlined in Section 7.

6.3 Dose Expansion Cohorts:

Once the maximum tolerated dose (MTD) is defined, an additional 6 to 9 patients will be treated at the selected highest dose level for a total 90 days (12-weeks) period to evaluate long-term safety and tolerability followed by a 7 to 10 days taper period to everyone on the study.

If 2 of the first 5 patients or if ≥ 2 of any 6 patients in this expansion cohort experience DLT, the Principal Investigator will discuss with all study investigators whether further addition of patients is needed to reassess the RP2D. Monitoring of all safety and toxicity data is done by the Principal Investigator, Co-Investigators and the whole study team.

6.4 Compliance with Oral Epidiolex

Compliance with oral Epidiolex will be documented by a medication diary and initial weight of study medication bottles as well as weight of returned study medication bottles by IDS study staff as per SOPs (CRO-PMC-C-015, CRO-PMC-C-014). See Appendix D for the IDS drug accountability form. Additionally, the patient will be requested to maintain a medication diary of each dose of medication. The medication diary will be returned to clinic staff at the end of each course, see Appendix E for patient medication tracking log and instructional handout for Epidiolex.

6.5 General Concomitant Medication and Supportive Care Guidelines

The clinical pharmacist reviews all concomitant medications at each clinic visit. Because there is a potential for interaction of Epidiolex with other concomitantly administered drugs, the case report form must capture the concurrent use of all other drugs, over-the-counter medications, or alternative therapies. The Principal Investigator or Co-investigators should be alerted if the patient is taking any agent known to affect or with the potential for drug interactions.

At the discretion of the treating investigator, concurrent administration of Epidiolex **is allowed with caution** with moderate or potent inhibitors/inducers of CYP1A2, CYP2B6, CYP2C8, CYP2C9, UGT1A9 and UGT2B7.

The use of moderate or potent inducers or inhibitors of CYP3A4 or CYP2C19 **are allowed with caution and consideration of Epidiolex dose reduction**. Lexicomp recommends dose adjustment of Epidiolex among participants using medications of these substrates; **Appendix C** provides a *partial* list of these medications. Please refer to the package insert for guidance.

Depression with suicidal ideation is a possible side effect of Epidiolex. Depression assessments will be performed using the validated National Comprehensive Cancer Network (NCCN) Distress Thermometer, as the institutional standard of care 10-point distress screening test performed at every clinic visit for every patient. Trained counselors are alerted to high distress scores, and evaluate these patients and provide counselling at the point-of-care and remain in direct contact with the care providers and team. See Section 7.2 for Epidiolex dose modifications/delays with distress scores and suicidal ideation.

6.6 Duration of Therapy

In the absence of treatment delays due to adverse event(s), treatment with Epidiolex may continue for 12 weeks or until one of the following criteria applies:

- Completion of study therapy
- Disease progression – Presence of any detectable distant metastases
- Symptomatic deterioration
- Intercurrent illness that prevents further administration of Epidiolex
- Patient decides to withdraw from the study
- Unacceptable toxicity
- Treatment delay of greater than 2 weeks
- Patients with signals of drug abuse or non-compliance
- General or specific changes in the patient's condition render the patient unacceptable for further

treatment in the judgment of the investigator
- Termination of the study by sponsor
- The drug manufacturer can no longer provide the study agent

The reason(s) for discontinuation of Epidiolex, the reason(s) for study removal, and the corresponding dates must be documented in the Case Report Form (eCRF).

6.7 Duration of Follow-Up

For those patients who completed the 90 days (12-weeks) study period or withdrawal from the study for any reasons should follow 10-day taper period to avoid withdrawal symptoms.

Patients will be followed for AEs for a total of 30 days after discontinuation (last dose) of Epidiolex.

Patients removed from the study treatment for unacceptable adverse event(s) will be followed until resolution or stabilization of the adverse event.

6.8 10-day Taper Dose Formula

For all patients, the MTD dose or the dose that patients last taken will be **tapered every 3 days** for total 7-10 days. Table below outlines examples of the appropriate taper for each dose level.

	**Taper for each dose level**			
**Starting Dose**	**Days 1 – 3**	**Days 4 - 6**	**Days 7 – 10**	**Day 11**
**Dose Level 2**<br>800mg per day	600mg once daily	400 mg once daily	200 mg once daily	Dose is stopped
**Dose Level 1**<br>600mg per day	400mg once daily	200 mg once daily	100 mg once daily	Dose is stopped
**Dose Level -1**<br>400mg per day	200mg once daily	100 mg once daily	Dose is stopped	Dose is stopped
**Dose Level -2**<br>200mg per day	100mg once daily	Dose is stopped	Dose is stopped	Dose is stopped

7. DOSE MODIFICATIONS AND DOSE DELAYS

7.1 Dose Delays/Modifications on Hepatotoxicity during Treatment

Abnormal LFTs	Management/Next dose for Epidiolex
AST and or ALT >1 time and normal total bilirubin	No change in dose
AST and or ALT > 3 times ULN and normal total bilirubin level	Hold until AST and or ALT < 1times ULN
AST and or ALT >2 times ULN and total bilirubin < 2 times ULN	Hold until AST and or ALT < 1 times ULN and normal total bilirubin level
AST and or ALT >3 times ULN and total bilirubin >2 times ULN	Discontinue treatment
Sustained AST and or ALT > 5 times ULN	Discontinue treatment

7.2 Dose Delays/Modifications on Suicidal Ideation during Treatment

	Management/Next dose for Epidiolex
**Distress Thermometer score ≥8**	Per Markey institutional guidelines, patient seen immediately in consultation by PsychoOncology Services for distress management. Potential treatment modification at the discretion of the treating investigator.
**Distress Thermometer score 7-4**	Per Markey institutional guidelines, patient is contacted by PsychoOncology Services for consultation. Potential treatment modification at the discretion of the treating investigator.
**Suicidal ideation present (Grade 2 or greater per CTCAE)**	Hold treatment and refer for consultation per institutional guidelines. Drug modification at the discretion of the treating investigator.

7.3 Dose Delays/Modifications for CNS toxicity, any Hematological toxicities (anemia, neutropenia, thrombocytopenia), any Gastrointestinal toxicities (diarrhea, nausea, vomiting) or others adverse events during treatment

CNS toxicity	Management/Next Dose for *EPIDIOLEX*
≤ Grade 1	No change in dose
Grade 2	Hold until ≤ Grade 1. Resume at same dose level.
Grade 3	Hold* until < Grade 2. Resume at one dose level lower, if indicated.**
Grade 4	Off protocol therapy

CNS toxicity	**Management/Next Dose for *EPIDIOLEX***
*Patients requiring a delay of >2 weeks should go off protocol therapy.<br>**Patients requiring >one dose reduction should go off protocol therapy.	

Anemia	**Management/Next Dose for *EPIDIOLEX***
≤ Grade 1	No change in dose
Grade 2	Hold until ≤ Grade 1. Resume at same dose level.
Grade 3	Hold* until < Grade 2. Resume at one dose level lower, if indicated.**
Grade 4	Off protocol therapy
*Patients requiring a delay of >2 weeks should go off protocol therapy.<br>**Patients requiring >one dose reduction should go off protocol therapy.	

Neutropenia	**Management/Next Dose for *EPIDIOLEX***
≤ Grade 1	No change in dose
Grade 2	Hold until ≤ Grade 1. Resume at same dose level.
Grade 3	Hold* until < Grade 2. Resume at one dose level lower, if indicated.**
Grade 4	Off protocol therapy
*Patients requiring a delay of >2 weeks should go off protocol therapy.<br>**Patients requiring >one dose reduction should go off protocol therapy.	

Thrombocytopenia	**Management/Next Dose for *EPIDIOLEX***
≤ Grade 1	No change in dose
Grade 2	Hold until ≤ Grade 1. Resume at same dose level.
Grade 3	Hold* until < Grade 2. Resume at one dose level lower, if indicated.**
Grade 4	Off protocol therapy
*Patients requiring a delay of >2 weeks should go off protocol therapy.<br>**Patients requiring >one dose reduction should go off protocol therapy.	

Diarrhea, Nausea or Vomiting	**Management/Next Dose for *EPIDIOLEX***
≤ Grade 1	No change in dose
Grade 2	Hold until ≤ Grade 1. Resume at same dose level.
Grade 3	Hold* until < Grade 2. Resume at one dose level lower, if indicated.**
Grade 4	Off protocol therapy
*Patients requiring a delay of >2 weeks should go off protocol therapy.<br>**Patients requiring >one dose reduction should go off protocol therapy.	

7.4 Other Grade 3 or 4 Non-hematologic toxicities

Hold all treatment until toxicities resolve to < Grade 2 and discuss with PI prior to restarting

8. PHARMACEUTICAL INFORMATION FOR EPIDIOLEX

8.1 Epidiolex Oral Solution, Mechanism of Action, PDs and PKs

Epidiolex oral solution is presented as an oily solution containing 100mg/ml. CBD dissolved in the excipients sesame oil and anhydrous ethanol with added sweetener (sucralose) and strawberry flavoring.

Formulation of Epidiolex Oral Solution	
Material	*Quantity*
CBD	100mg/ml
Anhydrous ethanol	79mg/ml
Sucralose	0.5 mg/ml
Strawberry flavoring	0.2 mg/ml
Sesame oil	Make up to 1ml

8.1.1 Mechanism of Action

The precise mechanisms by which Epidiolex exerts its anti-cancer effect in humans are unknown. Cannabidiol does not appear to exert its anticonvulsant effects through interaction with cannabinoid receptors as per the seizure study of pediatric population.

8.1.2 Pharmacodynamics. There are no relevant data on pharmacodynamic effects of cannabidiol.

8.1.3 Pharmacokinetics. Cannabidiol demonstrated an increase in exposure that was less than dose-proportional over the range of 5 to 20 mg/kg/day in patients.

8.2 Absorption, Distribution and Elimination of Epidiolex

Cannabidiol (Epidiolex oral solution) has a time to maximum plasma concentration (Tmax) of 2.5 to 5 hours at steady state (Css). Effect of food co-administration of EPIDIOLEX with a high-fat/high-calorie meal increased Cmax by 5-fold, AUC by 4-fold, and reduced the total variability, compared with the fasted state in healthy volunteers [see Dosage and Administration (2.2)].

Distribution. The apparent volume of distribution in healthy volunteers was 20,963 L to 42,849 L. Protein binding of the cannabidiol and its metabolites was >94% in vitro.

Elimination. The half-life of cannabidiol in plasma was 56- to 61-hours after twice-daily dosing for 7 days in healthy volunteers. The plasma clearance of cannabidiol following a single EPIDIOLEX 1500 mg dose (1.1 times the maximum recommended daily dosage) is 1111 L/h.

8.3 Metabolism and Excretion

Cannabidiol is metabolized in the liver and the gut (primarily in the liver) by CYP2C19 and CYP3A4 enzymes, and UGT1A7, UGT1A9, and UGT2B7 isoforms. After repeat dosing, the active metabolite of cannabidiol, 7-OH-CBD, has a 38% lower AUC than the parent drug. The 7-OH-CBD metabolite is converted to 7-COOH-CBD, which has an approximately 40-fold higher AUC than the parent drug. Based on preclinical models of seizure, the 7-OH-CBD metabolite is active; however, the 7-COOH-CBD metabolite is not active.

Excretion. EPIDIOLEX is excreted in feces, with minor renal clearance.

8.4 Specific Populations – Hepatic Impairment

Patients with Hepatic Impairment: No effects on the exposures of cannabidiol or metabolite exposures were observed following administration of a single dose of EPIDIOLEX 200 mg in patients with mild (Child-Pugh A) hepatic impairment.

Patients with moderate (Child-Pugh B) or severe (Child-Pugh C) hepatic impairment had an approximately 2.5 to 5.2-fold higher AUC, compared with healthy volunteers with normal hepatic function.

8.5 Special Considerations - Drug Accountability.

The investigator has overall responsibility for the accountability. The UK Investigational Drug Service (IDS) will maintain a careful record of the receipt; dispensing and final disposition of all Epidiolex received using the appropriate Investigational Drug Accountability Record form (DARF) – See Appendix D.

A list of the adverse events and potential risks associated with Epidiolex (the investigational agent administered in this study) can be found in Section 10.1.

8.6 Drug Interaction Studies, Transporters and In Vivo

In Vitro Assessment of Drug Interactions Drug Metabolizing Enzymes. Cannabidiol is a substrate for cytochrome p450 (CYP) enzymes CYP3A4 and CYP2C19. Cannabidiol has the potential to inhibit CYP2C8, CYP2C9, and CYP2C19 at clinically relevant concentrations. Cannabidiol may induce or inhibit CYP1A2 and CYP2B6 at clinically relevant concentrations. Cannabidiol inhibits uridine 5'-diphospho-glucuronosyltransferase (UGT) enzymes UGT1A9 and UGT2B7, but does not inhibit the UGT1A1, UGT1A3, UGT1A4, UGT1A6, or UGT2B17 isoforms.

Transporters. Cannabidiol and the cannabidiol metabolite, 7-OH-CBD, are not anticipated to interact with BCRP, BSEP, MDR1/P-pg, OAT1, OAT3, OCT1, OCT2, MATE1, MATE2-K, OATP1B1, or OATP1B3. The cannabidiol metabolite, 7-COOH-CBD, is not a substrate of BCRP, OATP1B1, OATP1B3, or OCT1. However, 7-COOH-CBD is a substrate for P-gp. 7-COOH-CBD is an inhibitor of transport mediated via BCRP and BSEP at clinically relevant concentrations.

In Vivo Assessment of Drug Interactions. Drug Interaction Studies with AEDs Clobazam and Valproate The interaction potential with other AEDs (clobazam and valproate) was evaluated in dedicated clinical studies following co-administration of EPIDIOLEX (750 mg twice daily in healthy volunteers and 20 mg/kg/day in patients). Co-administration with clobazam in healthy volunteers increased the cannabidiol active metabolite 7-OH CBD mean Cmax by 73% and AUC by 47%; and increased the clobazam active metabolite, N-desmethylclobazam, Cmax and AUC by approximately 3-fold [see Drug Interactions (7.2)]. When EPIDIOLEX was co-administered with valproate, there was no effect on valproate exposure. Effect of EPIDIOLEX on Midazolam Co-administration of EPIDIOLEX with midazolam (a sensitive CYP3A4 substrate) did not result in changes in plasma concentrations of midazolam compared to midazolam administered alone.

8.7 Supply, Storage and Handling of Epidiolex

8.7.1 How Supplied.

EPIDIOLEX is a strawberry flavored clear, colorless to yellow solution supplied in a 105 mL amber glass bottle with a child-resistant closure containing 100 mL of oral solution (NDC 70127-100-01). Each mL contains 100 mg of cannabidiol. EPIDIOLEX is packaged in a carton with two 5 mL calibrated oral dosing syringes and a bottle adapter (NDC 70127-100-10). The pharmacy will provide 1 mL calibrated oral dosing syringes when doses less than 1mL are required.

8.7.2 Agent Ordering and Procurement of medications:

Prescriptions for medications will be written by the site PI, treating physician, preferably the medical oncologist, using study-approved standardized Markey Cancer Center chemotherapy order sets. The University of Kentucky Investigational Drug Service will review and approve these orders per published polices. IDS will order and dispense study supply of commercially available EPIDIOLEX during the treatment phase of this trial. Drug accountability will be maintained on a National Cancer Institute Drug Accountability Report Form (DARF).

8.7.3 Storage and Handling

EPIDIOLEX in its original bottle in an upright position at 20°C to 25°C (68°F to 77°F); excursions are permitted between 15°C to 30°C (59°F to 86°F). [See USP Controlled Room Temperature]. Do not refrigerate or freeze. Keep the cap tightly closed. Use within 12 weeks of first opening the bottle, then bring all the empty bottles to clinic for check for compliance. The Epidiolex product is manufactured and packaged by GW Pharma Ltd (GWP). It will be distributed by our internal pharmacy. It must also be kept away from heat and direct sunlight.

The University of Kentucky Investigational Drug Service pharmacist will ensure that all study drug is stored in a secured, limited access storage area, under recommended storage conditions in accordance with applicable labeling and regulatory requirements and as provided by the separate study drug accountability manual. Under no circumstances should the investigator or other site personnel supply study drug to other investigators, patients, or clinics. Adequate records documenting receipts, use, return, loss, or other disposition of study provided drugs must be kept. The University of Kentucky Investigational Drug Service will supply drug accountability forms that will be used, or may approve use of standard institution forms. Drug accountability and supply order instructions and forms will be provided in a separate study manual. The accountability ledgers will be maintained to contain current and accurate inventory records and must be readily available for inspection. Unless otherwise authorized by the sponsor, at the end of the clinical trial all drug supplies unallocated or unused by sites or patients must be returned to the University of Kentucky Investigational Drug Service (UK IDS) for final actions in accordance with sponsor instructions.

9. STATISTICAL CONSIDERATIONS

9.1 Study Design/Endpoints

This is a phase I dose escalation study with expansion cohort. Dose escalation will be determined by a Bayesian optimal interval (BOIN) design [41]. The target DLT rate is 30%. Patients are enrolled in cohorts with 3 patients in each cohort. Enrollment will be temporarily halted after the last patient of each cohort is enrolled until all current patients are DLT evaluable or withdrawn from the study. A patient will be considered DLT evaluable if he completes at least 75% of the planned dose of Epidiolex in the first 30 days of the treatment, unless the reason is due to toxicity.

The primary objective is to evaluate the acute toxicity and long-term safety and tolerability of Epidiolex (CBD) in patients with biochemically recurrent prostate cancer. Acute toxicity of Epidiolex is evaluated through DLT rate, which is calculated as the total number of patients experienced DLTs at a dose level divided by the total number of patients treated at the corresponding dose level. The calculation of DLT rate will only include DLT evaluable patients. Patients who are not DLT-evaluable will not be replaced for the current cohort and will not be included in the calculation of DLT rate. At the end of the dose escalation part of the study, we will use the dose escalation/de-escalation decision rules (see section 9.1.1) to decide the dose level for dose expansion cohort. If the current dose at the end of dose escalation is already the highest available dose, and the decision rules did not indicate de-escalation or elimination, the highest dose level is deemed as safe for acute toxicity; otherwise, the dose recommended by the decision rule is the estimated MTD.

Following dose-escalation, an expansion cohort will be enrolled to confirm safety for acute toxicity, evaluate long-term safety and tolerability and explore evidence of efficacy of the study treatment. Long-term safety and tolerability are evaluated through the summary statistics of adverse events occurred within the 90-day follow-up period in patients who received any amount of study drug. The study treatment is deemed as not safe for long-term tolerability if any of the following criteria are met:

- More than 40% of the patients treated at the dose level selected for expansion cohort experienced grade 3 or higher AEs that are possibly/probably/definitely related to study treatment
- More than 30 % of the patients experienced grade 3 or higher CNS toxicity that are possibly/probably/definitely related to study treatment

9.1.1 Dose Escalation / De-escalation Decision Rule

	**Number of patients Treated**								
**Decision Rule**	**1**	**2**	**3**	**4**	**5**	**6**	**7**	**8**	**9**
Escalate (or highest) if # DLT <=	0	0	0	0	1	1	1	1	2
De-escalate (or lowest) if # DLT >=	1	1	2	2	2	3	3	3	4
Eliminate (or stop) if # DLT >=	--	--	3	3	4	4	5	5	5

Note: the next cohort will be treated at the current dose level if the criteria are not met for escalation/de-escalation rules.

9.1.2 Secondary and Correlative Endpoints

The secondary endpoints include change in serial PSA, PSA velocity and testosterone levels from baseline

throughout the treatment period as an indication of biochemical response and health-related quality of life (as assessed by the EORTC QLQ-C30 and QLQ-PR25).

The correlative endpoint includes CBD receptor 1 and 2 expression levels.

9.2 Sample Size/Accrual Rate

We plan to enroll up to 9 DLT evaluable patients for dose escalation. If 3 or more of the first 9 patients are not evaluable for DLT, additional cohort(s) will be enrolled to ensure at least 9 DLT evaluable patients for dose escalation. Once we have established the highest tolerated dose is deemed as acceptable for acute toxicity or estimated the MTD, we will begin treating 6 to 9 more patients in an expansion cohort to confirm safety for acute toxicity, evaluate long-term tolerability and explore evidence of efficacy of the study treatment. The number of patients to be enrolled in expansion cohort depends on the DLT rate during the dose escalation part of the study. The total planned sample size for this study is 18 (enrolled) patients with about 12 patients treated at the MTD or the highest planned dose.

9.3 Analysis of Primary Endpoint

Dose escalation decisions and MTD are estimated through the rules specified in section 9.1.1. DLT rate at each dose level will be calculated along with the Fisher's exact confidence interval at 95% confidence level. AE, SAE and AE of special interests will be summarized by descriptive statistics to evaluate long-term safety.

9.4 Analysis of Secondary Endpoints

Change in serial PSA, PSA velocity and testosterone levels from baseline throughout the 90-day treatment period will be represented by longitudinal profiles and analyzed by mixed effects model. Baseline is defined as the last non-missing PSA measurement prior to administrating Epidiolex. PSA and testosterone levels may be categorized and summarized by response rates with confidence intervals. Patients who are DLT evaluable, have baseline measurement and have at least one post-baseline measurement will be included in the analysis of secondary endpoints.

The health-related quality of Life (EORTC QLQ-C30 and QLQ-PR25) collected longitudinally will be analyzed using appropriate linear models for repeated measures data.

9.5 Analysis of Correlative Endpoints

Post-hoc analyses will be conducted on correlative endpoints with appropriate statistical methods depending on distribution and availability of collected data.

10. ADVERSE EVENTS: LIST AND REPORTING REQUIREMENTS

Adverse event (AE) monitoring and reporting is a routine part of every clinical trial. The following lists of AEs (Section 10.1) and the characteristics of an observed AE (Sections 10.2 and 10.3) will determine whether the event requires expedited reporting **in addition** to routine reporting.

10.1 Adverse Event List for Epidiolex

The most common adverse reactions (10% or more) for Epidiolex are:

- Somnolence
- Decreased appetite
- Diarrhea
- Transaminase elevations
- Fatigue
- Malaise
- Asthenia
- Rash
- Infection
- Insomnia, sleep disorder and poor-quality sleep. The patient will be asked "On a scale of 0 to 10, please indicate the number best describes your/the patients sleep disruption in the last week." The markers range from 0= slept extremely well, to 10= unable to sleep at all.
- Mood changes including depressive symptomatology and Suicidal Ideation (*less common*)

  An AE that is consistent with the above categories will be known as a "triggering AE of interest" for the purpose of this study.

10.1.1 Monitoring of Drug Abuse Liability

Two triggers will require the investigator or study coordinator to discuss abuse potential signals with the patient. These comprise *AEs of interest* that may be reported by the patient/caregiver, **or** drug accountability issues regarding overuse of the Epidiolex or missing bottles.

The categories for **triggering AEs of interest** are:

- Euphoria or inappropriate elation
- Inappropriate laughter or exhilaration
- Mood changes
- Drunk, high or intoxicated
- Hallucinations (visual or auditory), dissociations, disorientation, agitation.
- Disturbance in cognition, memory, or attention
- Drug abuse
- Drug withdrawal or drug withdrawal syndrome
- Addiction
- Overdose
- Misuse of Epidiolex
- **Thoughts of suicide, attempted suicide or suicide**

10.1.2 List of "Triggering Drug Accountability Discrepancies"

If there are any discrepancies in drug accountability as outlined by the criteria below, known as "triggering drug accountability discrepancies", then the investigator or study coordinator will complete a Supplemental Drug Accountability Form. The team (investigator, treating physician and study staff) will conduct further discussion of the event, which will be addressed at a later time with the patient.

The triggering drug accountability discrepancies are as follows:

- Missing bottle(s).
- Compliance issues where one or more bottles are used, compared to the expected use.
- Returned Epidiolex supply with evidence of tampering.
- Greater than the target daily dose as instructed.

10.2 Adverse Event characteristics

- **CTCAE term (AE description) and grade:**
  CTCAE term (AE description) and grade: The descriptions and grading scales found in the revised NCI Common Terminology Criteria for Adverse Events (CTCAE) version 5.0 will be utilized for AE reporting. All appropriate treatment areas have access to a copy of the CTCAE version 5.0. The CTCAE version 5.0 can be downloaded from CTEP web site: https://ctep.cancer.gov/protocoldevelopment/electronic_applications/docs/CTCAE_v5_Quick_Reference_8.5x11.pdf

- **For expedited reporting purposes only:**
  -AEs for Epidiolex listed above in 10.1 and 10.1.2 should be reported only if the adverse event varies in nature, intensity or frequently from the expected toxicity information.

- **Attribution** of the AE:
  - Definite – The AE is *clearly related* to the study treatment
  - Probable -- The AE is *likely related* to the study treatment
  - Possible – The AE *may be related* to the study treatment
  - Unlikely – The AE is *doubtfully related* to the study treatment
  - Unrelated – The AE is *clearly NOT related* to the study treatment

10.3 MCC Expedited adverse event reporting

10.3.1 For MCC Investigator-Initiated Trials (IITs), investigators must report to the Overall PI any serious adverse event (SAE) that occurs after the initial dose of study treatment, during treatment, or within 30 days of the last dose of study treatment on the local institutional SAE form. This applies only to the following categories:

- **Grade 2 or greater suicidal ideation,** ***as assessed per Markey institutional guidelines for identification and triage of distress and graded per the CTCAE v5.0 as follows:,***
  Grade 2 = current suicidal ideation with no plan or intent
  Grade 3 = specific plan to commit suicide without serious intent to die which may not require hospitalization
  Grade 4 = specific plan to commit suicide with serious intent which requires hospitalization

- **Grade 3 (severe)** ***Medical*** **Events** – Expedited reporting for events that are Unexpected and *Possibly, Probably or Definitely* Related/Associated with Epidiolex

- **ALL Grade 4 (life threatening or disabling)** ***Medical*** **Events** – Expedited Reporting of the event, Unless expected AND specifically listed in protocol as not requiring expedited reporting.

- **ALL Grade 5 (fatal) Events**, report all events regardless of study phase or attribution

Note: Abnormal laboratory values are not considered medical events, unless determined to be causative of SAE by the investigator or Grade 5.

10.3.2 Timeframes for Expedited Reporting for Markey IITs

Investigators within MCC will report SAEs directly to Markey's Data and Safety Monitoring Committee per the DSMC's SOP, and also to the University of Kentucky Institutional Review Board per IRB reporting policies. Use the MCC protocol number and the protocol-specific patient ID assigned during trial registration on all reports.

Note: A death on study requires both routine and expedited reporting, regardless of causality. Attribution to treatment or other cause must be provided.

Death due to progressive disease should be reported as Grade 5 "Disease progression" in the system organ class (SOC) "General disorders and administration site conditions." Evidence that the death was a manifestation of underlying disease (e.g., radiological changes suggesting tumor growth or progression: clinical deterioration associated with a disease process) should be submitted.

Phase 1 and Early Phase 2 Studies: Expedited Reporting Requirements for Adverse Events that Occur within 30 Days of the Last Administration of Epidiolex [1, 2]

FDA REPORTING REQUIREMENTS FOR SERIOUS ADVERSE EVENTS (21 CFR Part 312)
NOTE: Investigators **MUST** immediately report to the sponsor **ANY** Serious Adverse Events, whether or not they are considered related to the investigational agent(s)/intervention (21 CFR 312.64)
An adverse event is considered serious if it results in **ANY** of the following outcomes:

1) Death
2) A life-threatening adverse event
3) An adverse event that results in inpatient hospitalization or prolongation of existing hospitalization for ≥ 24 hours
4) A persistent or significant incapacity or substantial disruption of the ability to conduct normal life functions
5) A congenital anomaly/birth defect.
6) Important Medical Events (IME) that may not result in death, be life threatening, or require hospitalization may be considered serious when, based upon medical judgment, they may jeopardize the patient or subject and may require medical or surgical intervention to prevent one of the outcomes listed in this definition. (FDA, 21 CFR 312.32; ICH E2A and ICH E6).

ALL SERIOUS adverse events that meet the above criteria MUST be immediately reported via electronic submission within the timeframes detailed in the table below.

Hospitalization	Grade 1 and Grade 2 Timeframes	Grade 3-5 Timeframes
Resulting in Hospitalization ≥ 24 hrs	10 Calendar Days	24-Hour 5 Calendar Days
Not resulting in Hospitalization ≥ 24 hrs	Not required	

NOTE: Protocol specific exceptions to expedited reporting of serious adverse events are found in the Specific Protocol Exceptions to Expedited Reporting Section.

Expedited AE reporting timelines are defined as:

- "24-Hour; 5 Calendar Days" - The AE must initially be submitted electronically within 24 hours of learning of the AE, followed by a complete expedited report within 5 calendar days of the initial 24-hour report.
- "10 Calendar Days" - A complete expedited report on the AE must be submitted electronically within 10 calendar days of learning of the AE.

[1]Serious adverse events that occur more than 30 days after the last administration of investigational agent/intervention and have an attribution of possible, probable, or definite require reporting as follows:
Expedited 24-hour notification followed by complete report within 5 calendar days for:

- All Grade 3, 4, and Grade 5 AEs

Expedited 10 calendar day reports for:

- Grade 2 AEs resulting in hospitalization or prolongation of hospitalization

[2]For studies using PET or SPECT IND agents, the AE reporting period is limited to 10 radioactive half-lives, rounded UP to the nearest whole day, after the agent/intervention was last administered. Footnote "1" above applies after this reporting period.

10.4 Expedited reporting to UKHC Hospital Risk Management

Participating investigators will report to the UK Office of Risk Management any participant safety reports or sentinel events that require reporting according to institutional policy.

10.5 Routine adverse event reporting

All Adverse Events must be reported in routine study data submissions to the Overall PI on the OnCore case report forms. **AEs reported expeditiously to the Overall PI and DSMC via OnCore must also be reported in routine study data submissions.**

10.6 Secondary Malignancy

A *secondary malignancy* is a cancer caused by treatment for a previous malignancy (*e.g.*, treatment with investigational agent/intervention, radiation or chemotherapy). A secondary malignancy is not considered a metastasis of the initial neoplasm.

All secondary malignancies that occur following treatment with an agent under an NCI IND/IDE must be reported to overall PI and DSMC and Office of Research Integrity, as well the FDA and sponsor in according to reporting requirements. Three options are available to describe the event:

- Leukemia secondary to oncology chemotherapy (*e.g.*, acute myelocytic leukemia [AML])
- Myelodysplastic syndrome (MDS)
- Treatment-related secondary malignancy

Any malignancy possibly related to cancer treatment (including AML/MDS) should also be reported via the routine reporting mechanisms outlined in each protocol.

10.7 Second Malignancy

A *second* malignancy is one unrelated to the treatment of a prior malignancy (and is **NOT** a metastasis from the initial malignancy). Second malignancies require **ONLY** routine AE reporting unless otherwise specified.

11. STUDY CALENDAR

Baseline evaluations including labs and urine studies are to be conducted within 30 days prior to start of investigational therapy. Scans and x-rays must be done within 12 weeks prior to the start of therapy.

Procedures	Pre-study Screening	Day 1 CBD administration	Day 30 post-CBD initiation	Day 60 post-CBD initiation	Day 90 post-CBD initiation	30-days post final CBD dose	Off-Study
Epidiolex (CBD), oral solution [1]		X[1a]	X	X	X		
Adverse Event Evaluation			X	X	X	X	
Assess dose-limiting toxicities			X	X	X		
Assess CBD oral compliance [2]			X	X	X		
Quality of Life [3]	X				X		
Informed Consent	X						
Demographics	X						
PSA (serum, one tube) [4]	X		X	X	X	X	
Total Testosterone [5]	X		X	X	X	X	
Fluciclovine PET or PET/CT scan [6a]	X*,**	Scans are repeated per NCCN guidelines, or more often, as clinically indicated by suspected disease progression.					
Bone scan [6a]	X*,**						
CT chest, abdomen, pelvis [6a]	X*,**						
MRI of pelvis [6b]	X*						
Assessment of Disease Progression [6c]	X	X	X	X	X	X	X
Urine THC screen [7]	X						
EKG	X		X	X	X		
CBC w/ diff, platelets [8]	X	X	X	X	X	**	
CMP [9]	X	X	X	X	X	**	
Concomitant Medications	X		X	X	X		
Medical History [11]	X						
Height	X						
Physical Exam with vitals (including weight) [12]	X	**	X	X	X	**	
ECOG Performance Status	X	**	X	X	X	**	
Archival tissue collection, if available	X	If archival tissue is available, it will be collected for testing of CB1 and CB2 receptors by IHC (correlative endpoint).					

Procedures	Pre-study Screening	Day 1 CBD administration	Day 30 post-CBD initiation	Day 60 post-CBD initiation	Day 90 post-CBD initiation	30-days post final CBD dose	Off-Study
Record date of ADT initiation [10]		ADT initiation renders a patient ineligible; study staff will record date in the eCRF and take the patient off-study treatment.					

NOTES:
* per standard of care
** at the discretion of the treating physician.

1: Epidiolex (CBD) is an oral solution (taken by mouth), on an outpatient basis, dose as assigned.
1a: Within 24-hours of Day 1 Epidiolex administration, Study Coordinator will do a follow-up visit via phone with participants in order to assess compliance and trouble-shoot issues with Epidiolex administration at home, and also to reinforce completion of patient diary (medication tracking log, Appendix E). This Day 2 follow-up phone visit has a 3-day window to avoid deviations, **however** EVERY EFFORT will be made to do this check-in within 24-hours of the initial dose on Day 1.

2: Oral compliance for the Epidiolex (CBD) will be conducted by study staff. Compliance will be documented by a medication diary and bottle weights at initiation and at return of study medication bottles by study staff. Patients will be requested to maintain a medication diary of each dose of medication. The medication diary will be returned to clinical staff at the end of each course.)

3 : QOL is assessed via EORTC QLQ-C30 + QLQ-PR25 at 2 timepoints
- at a clinic visit prior to first dose of Epidiolex
- at a clinic visit; either the end of Epidiolex administration OR when Epidiolex is discontinued (if stop it early). The final QOL timepoint can be +/- 3 weeks, and conducted during a regularly scheduled, standard of care clinic visit.

4, 5: One tube of blood will be drawn at each of the noted timepoints for serum PSA and total testosterone; this is standard of care for men with rising PSA after treatment for prostate cancer, baseline and every 3 months. Additional blood draws for the PSA and testosterone during Epidiolex administration may be within a + 7-day window to align with a scheduled clinic visit.

6: *Imaging at baseline, pre-study*: Patients who have confirmed recurrent disease of the prostate are deemed ineligible for this trial. As such, all eligible patients will undergo standard of care imaging to identify metastases (initial evaluation of status/extent of recurrent disease), including:
6a: All eligible patients will undergo standard of care imaging to assess extent of metastatic disease at baseline; fluciclovine PET scan is preferred; other scans (PET/CT scan, bone scan and/or CT of the chest, abdomen and pelvis) may be performed as needed at baseline to assess extent of metastatic disease, per standard of care. Axium PET/CT as clinically appropriate.
6b: *Additionally*, eligible patients who have undergone radiation prior to study enrollment will have an MRI of pelvis (in addition to previously named imaging studies) to assess for recurrent local disease. Suspicious lesions of the prostate seen on MRI will be biopsied.
6c: *Imaging on Study as work-up for disease progression*: Once a patient is enrolled on-study, imaging to assess disease progression will proceed per standard of care, based on clinical indicators such as rising PSA and/or other clinical symptoms. Re-evaluation of potential metastases is at the discretion of the treating physician based on clinical indicators. Notably, a rising PSA and/or other clinical symptoms could trigger scan/imaging.

Procedures	Pre-study Screening	Day 1 CBD administration	Day 30 post-CBD initiation	Day 60 post-CBD initiation	Day 90 post-CBD initiation	30-days post final CBD dose	Off-Study
**7:** Urine screen for THC may be repeated if patient's baseline screen is positive, triggering a one-week washout period before the patient can be assessed for eligibility status. Additionally, urine THC screen may be repeated at the discretion of investigator if suspected use by patient while on-study.<br><br>**8:** CBC with diff is routine blood test conducted at clinic visits; only, white count, hemoglobin, hematocrit, and platelets count and WBC differential counts if abnormal WBC will be recorded in the eCRF.<br><br>**9:** CMP is routine blood test during clinic visits and will be done at the discretion of the treating physician; alkaline phosphatase, total bilirubin, creatinine, glucose, electrolytes, SGOT [AST] will be recorded in the eCRF.<br><br>**10:** If a patient on-study initiates androgen deprivation therapy of any kind, he is ineligible and will be removed from the study. Study staff will monitor this and record date of initiation of ADT, should that event occur.<br><br>**11:** Full Medical History, also specifically assess for history of depression and suicidal ideation/attempt.<br><br>**12:** Physical Exam now includes assessment for new onset depressive symptoms and/or suicidal ideation as a potential treatment-related adverse event. Per Markey institutional guidelines, patients complete Distress Thermometer at each visit, high distress scores trigger consultation with PsychoOncology; for any such consultation, the treating physician and the Principal Investigator will be informed of the results as regards new onset suicidal ideation/attempt or other depressive symptoms; Epidiolex treatment will be modified at the discretion of the treating investigator.							

12. MEASUREMENT OF EFFECT

Although the clinical benefit of Epidiolex has not yet been established, the intent of offering this treatment is to provide a possible therapeutic benefit, and thus the patient will be carefully monitored for PSA response and symptom relief in addition to safety and tolerability. For the purposes of this study, patients should be re-evaluated every 4 weeks during the 90-day study duration. In addition to a baseline PSA level, serial PSA levels will be obtained every 4 weeks during the study period.

12.1 Antitumor Effect – Solid Tumors

12.1.1 Response Criteria

Biochemical response will be determined by measurement of PSA approximately every 4 weeks during treatment.

Complete Biochemical Response: Normalization of PSA (PSA level becomes undetectable) documented by two different measurements taken at least 4 weeks apart in patients with prior radiation therapy and an undetectable PSA on two consecutive measurements taken at least 4 weeks apart in patients with a prior surgery.

Partial Biochemical Response: Reduction in PSA by ≥ 25% (from baseline) documented by two different measurements taken at least 4 weeks apart.

PSA Progression: An increase in baseline PSA ≥ 50% documented by two different measurements taken at least 4 weeks apart.

Clinical Progression: Any positive metastases findings on bone scan and/or CT scan of the abdomen or pelvis or Axium PET/CT scan or development of symptoms attributable to cancer progression.

Stable Biochemical Disease: An increase in baseline PSA <25% documented by two different measurements taken at least 4 weeks apart.

12.1.2 Duration of Response

Duration of overall response: The duration of overall response is measured from the time measurement criteria are met for biochemical response (complete or partial or stable whichever the first occurrence) until the first date that clinical progression disease is objectively documented, up to 30-days after the last dose of the planned 90-day administration of Epidiolex.

12.2 Health-related Quality of Life

HRQOL will be reported by participants using the EORTC core quality of life questionnaire (QLQ-C30) and prostate cancer specific module (QLQ-PR25). (See Appendix B) HRQOL is a secondary outcome in this trial and the specific HRQOL objective is to determine whether the investigational agent has an impact on improvement in quality of life. The QLQ-C30 is a validated questionnaire developed to assess HRQOL in cancer patients. It includes five functional scales (physical, role, cognitive, emotional, and social), three symptom scales (fatigue, pain, and nausea and vomiting), and a global health and quality-of-life scale. The remaining single items assess additional symptoms commonly reported by cancer patients (dyspnea, appetite loss, sleep disturbance, constipation, and diarrhea), as well as the perceived

financial impact of the disease and treatment [25, 40]. The QLQ-PR25 is a 25-item module that complements the QLQ-C30, and is designed to assess HRQOL in prostate cancer patients. It includes 5 multi-item scales assessing urinary symptoms, bowel symptoms, sexual activity, sexual function, and incontinence aids [26, 41].

13. STUDY OVERSIGHT, DATA REPORTING / REGULATORY REQUIREMENTS

13.1 Study Oversight

This protocol is monitored at several levels, as described in this section. The Protocol Principal Investigator is responsible for monitoring the conduct and progress of the clinical trial, including the ongoing review of accrual, patient-specific clinical and laboratory data, and routine and serious adverse events; reporting of expedited adverse events; and accumulation of reported adverse events from other trials testing the same drug(s). The Protocol Principal Investigator and statistician have access to the data at all times through the OnCore.

13.2 Protocol Review and Monitoring Committee and Institutional Review Board Review

Before implementing this study, the protocol must be reviewed by the Markey Cancer Center's Protocol Review and Monitoring Committee and the protocol, the proposed informed consent form and other information to subjects, must be reviewed by the University of Kentucky Institutional Review Board (IRB). A signed and dated UK IRB initial review approval memo must be maintained in the Markey Cancer Center Clinical Research Office (MCC CRO) regulatory binder. Any amendments to the protocol, other than administrative ones, must be reviewed and approved by the PRMC, study sponsor and the UK IRB.

13.3 Quality Assurance

The MCC places the highest priority on ensuring the safety of subjects participating in clinical trials and on the quality of data obtained from clinical and translation research. The MCC Quality Assurance (QA) Office oversees the maintenance of quality standards in clinical cancer research through clinical data monitoring of Investigator Initiated Trials (IITs) and routine audits.

13.3.1 Data Monitoring

The MCC QA Office will collaborate with the PI, Biostatisticians and Lead OnCore® Data Management Specialist in creating a Clinical Data Monitoring Plan (CDMP) using a risk-based approach. The CDMP will describe the scope, communication plan, and frequency of monitoring visits. In addition, describe query submissions and resolutions, action items and monitoring reports.

The QA monitor assigned to the trial will perform the monitoring tasks in accordance with the protocol specific CDMP. The monitoring process will provide research staff and PI with the opportunity to evaluate the progress of the study, verify the accuracy and completeness of the case report forms, assure that all protocol requirements, including applicable regulations and investigator's obligations are being fulfilled, and prompt resolution of any inconsistencies in the study records.

13.3.2 Audit

To ensure compliance with the International Conference on Harmonisation of Good Clinical Practice Guidelines and all applicable regulatory requirements, the MCC Audit Committee will conduct a quality assurance audit. A minimum of 25% of patients enrolled in the study may be selected for review. The purpose of a MCC audit is to systematically and independently examine all study-related activities and documents to determine whether these activities were conducted, and data were recorded, analyzed, and accurately reported according to the protocol, Good Clinical Practice guidelines of the International Conference on Harmonization, and any applicable regulatory requirements.

13.4 Data and Safety Monitoring Committee

The MCC Data and Safety Monitoring Committee (DSMC) will oversee the conduct of this trial. The MCC DSMC performs routine real-time data monitoring and safety review of all trials, with a special focus upon investigator-initiated trials (IITs). The MCC DSMC will conduct review of the trial on a schedule determined the MCC Protocol Review & Monitoring Committee (PRMC). The MCC DSMC will monitor the following elements of the trial: adverse event analysis, serious adverse events, protocol deviations/violations, and accrual. In addition, when applicable the MCC DSMC will review QA audits and monitoring reports, previous reviews by the DSMC, suggested actions by other committees, such as the IRB, UK Risk Management Committee, and other parameters and outcomes as determined by the DSMC. If appropriate, the DSMC will designate and monitor corrective action(s) based on review outcome. The MCC DSMC has the authority to amend, temporarily suspend, or terminate the trial based upon patient safety or compliance matters.

13.5 Data Reporting

13.5.1 Method

This study will require data submission and reporting via the OnCore Enterprise Research Clinical Trials Management System, which is the official database of the Markey Cancer Center. Instructions for submitting data is listed in study-specific guidance documents authored by a member of the MCC Data Management Team. These guidance documents may include any of the following, as appropriate for the scope of the study: eCRF Completion Guidelines, Data Management Specifications, Subject Console Guide, and Query Resolution Guide. These guidance documents will be approved and housed within OnCore to ensure access to approved versions to facilitate data submission.

13.5.2 Responsibility for Data Submission

This trial will be monitored by the MCC Data and Safety Monitoring Committee (DSMC) on a schedule determined by the Protocol Review and Monitoring Committee at the initial PRMC review. Study staff are responsible for submitting study data and/or data forms to OnCore as per the Markey Cancer Center SOPs. Study staff are responsible for compiling and submitting data for all participants and for providing the data to the Principal Investigator for review.

13.6 Data Management

Data management will be performed by cross-team members at MCC. These team members will include representatives from the Data Management Team, Biostatistics and Bioinformatics SRF, and the Quality Assurance Office. They will work closely with study staff to ensure timely and accurate data submission. A protocol-specific Data Management Plan (DMP) will be authored by a senior data manager in collaboration with the biostatistician and Principal Investigator with each expected to review and approve

the finalization of the DMP. In order to maintain best clinical practices in data management, the DMP may include, but not be limited to CRF/eCRF design, database build and design, database training, edit check/validation specifications, study database testing/release, data and paper workflow, report, metrics, query/discrepancy management, management of external (including lab) data, medical coding, SAE handling/reconciliation, data transfers and database lock. The protocol-specific DMP will additionally define the schedule at which data will be accessed by data management and study statistician to perform statistical programming for conduct of data quality, data control, data management, generation of interim reports and statistical analysis. Cross-team members will collaborate to establish procedures and timelines for quality control, audits, query resolution, annual reports, interim analysis and final data analysis.

13.7 Compliance with Laws and Regulations

The study will be conducted in accordance with U.S. Food and Drug Administration (FDA) and International Conference on Harmonization (ICH) Guidelines for Good Clinical Practice (GCP), the Declaration of Helsinki, any applicable local health authority, and Institutional Review Board (IRB) requirements. The PI or designee will be responsible for obtaining continuing and not less than annual IRB re-approval throughout the duration of the study. Copies of the Investigator's annual report to the IRB and copies of the IRB continuance of approval must maintained by the MCC CRO. The PI or designee is also responsible for notifying the Data and Safety Monitoring Committee of the MCC and the UK IRB of any significant adverse events that are serious and/or unexpected, as per SOP's of those entities and compliance with protocol requirements. The MCC DSMC will review all adverse events of this IIT as per its SOP.

14. REFERENCES

1. Dess RT, Morgan TM, Nguyen PL, et al. Adjuvant versus early salvage radiation therapy following radical prostatectomy for men with localized prostate cancer. Curr Urol Rep. 2017;18:55
2. Han M, Partin AE, Pound CR, et al. Long-term biochemical disease-free and cancer-specific survival following anatomic radical retropubic prostatectomy. The 15-year Johns Hopkins experience. Urol Clin North Am 2001;28:555
3. Cookson MS, Aus G, Burnett AL, et al. Variation in the definition of biochemical recurrence in patients treated for localized prostate cancer: The American Urological Association Prostate Guidelines for Localized Prostate Cancer Update Panel report and recommendations for a standard in the reporting of surgical outcomes. J Urol 2007;177:540
4. Freedland SJ, Humphreys EB, Mangold LA, et al. Risk of prostate cancer-specific mortality following biochemical recurrence after radical prostatectomy. JAMA 2005;294:433
5. Caire AA, Sun L, Ode O, et al. Delayed prostate-specific antigen recurrence after radical prostatectomy: how to identify and what are their clinical outcomes? Urology 2009;74:643-647.
6. D'Amico AV, Moul JW, Carroll PR, et al. Surrogate end point for prostate cancer-specific mortality after radical prostatectomy or radiation therpay. J Natl Cancer Inst 2003;95:1376
7. Pound CR< Partin AW, Eisenberger MA, et al. Natural history of progression after PSA elevation following radical prostatectomy. JAMA 1999;281:1591
8. Pinover WH, Horwitz EM, Hanlon AL, et al. Validation of a treatment policy for patients with prostate specific antigen failure after three dimensional conformal prostate radiation therapy. Cancer 2003;97:1127
9. Zagars GK, Pollack A. Kinetics of serum prostate-specific antigen after external beam radiation for clinically localized prostate cancer. Radiother Oncol 1997;44;213
10. Patel A, Dorey F, Franklin J, et al. Recurrence patterns after radical retropubic prostatectomy: Clinical usefulness of prostate specific antigen doubling times and log slope prostate specific antigen. J Urol 2003;170:1872
11. Loblaw DA, Virgo KS, Nam R, et al. Initial hormonal management of androgen-sensitive metastatic, recurrent, or progressive prostate cancer: 2006 update of an American Society of Clinical Oncology practice guideline. J Clin Oncol 2007;25:1596
12. Byar DP, Corle DK. Hormone therapy for prostate cancer: results of the Veterans Administration Cooperative Urological Research Group studies. NCI Monogr 1988:165
13. Moul JW, Wu H, Sun L, et al. Early verssu delayed hormonal therapy for prostate specific antigen only recurrence of prostate cancer after radical prostatectomy. J Urol 2004;171:1141]
14. Garcia-Albeniz X, Chan JM, Paciorek A, et al. Immediate versus deferred initiation of androgen deprivation therapy in prostate cancer patients with PSA-only relapse. An observational follow-up study. Eur J Cancer. 2015;51:817-824
15. McLeod DG, Iversen P, See WA, et al. Bicalutamide 150mg plus standard care vs. standard care alone for early prostate cancer. BJU Int 2006;97:247
16. Zareba P, Duivenvoorden W, Leong DP, Pinthus JH. Androgen deprivation therapy and cardiovascular disease: what is the linking mechanism? Ther Adv Urol. 2016 Apr;8(20:118-129.
17. Pruthi RS, Derksen JE, Moore D, et al. Phase II trial of celecoxib in prostate-specific antigen recurrent prostate cancer after definitive radiation therapy or radical prostatectomy. Clin Cancer Res 2006;12(7pt1):2172

18. Carmody J, Olendzki B, Reed G, et al. A dietary intervention for recurrent prostate cancer after definitive primary treatment: Results of a randomized pilot trial. Urology 2008;72:1324.
19. Schroder Fh, Roobol MJ, Boeve ER, et al. Randomized, double-blind, placebo-controlled crossover study in men with prostate cancer and risking PSA: Effectiveness of a dietary supplement. Eur Urol 2005;48:922
20. National Comprehensive Cancer Network, Prostate Cancer (Version 4.2019) https://www.nccn.org/professionals/physician_gls/pdf/prostate.pdf
21. Sytsma, K. J., et al. (2002). "Urticalean rosids: circumscription, rosid ancestry, and phylogenetics based on rbcL, trnL-F, and ndhF sequences." Am J Bot **89**(9): 1531-1546
22. Matsuda, L. A., et al. (1990). "Structure of a cannabinoid receptor and functional expression of the cloned cDNA." Nature **346**(6284): 561-564.
23. Munro, S., et al. (1993). "Molecular characterization of a peripheral receptor for cannabinoids." Nature **365**(6441): 61-65.
24. Bisogno T, Hanus L, De Petrocellis L, Tchilibon S, Ponde DE, Brandi I et al. Molecular targets for cannabidiol and its synthetic analogues: effect on vanilloid VR1 receptors and on the cellular uptake and enzymatic hydrolysis of anandamide. Br J Pharmacol. 2001 Oct;134(4):845–52.
25. Whyte LS, Ryberg E, Sims NA, Ridge SA, Mackie K, Greasley PJ et al. The putative cannabinoid receptor GPR55 affects osteoclast function in vitro and bone mass in vivo. Proc Natl Acad Sci U S A 2009;106(38):16511–6.
26. Pertwee RG. The pharmacology and therapeutic potential of cannabidiol. In: Di Marzo V, editor. Cannabinoids. New York: Kluwer Academic/Plenum publishers; 2004. p. 32–83.
27. Zuardi AW, Morais SL, Guimarães FS, Mechoulam R. Antipsychotic effect of cannabidiol. J Clin Psychiatry 1995;56(10):485–6.
28. Thiele EA, Marsh ED, French JA et al. Cannabidiol in patients with seizures associated with Leenox-Gastaut syndrome (GWPCARE4): a randomised, double-blind, placebo-controlled phase 3 trial. Lancet 2018 Mar 17;391
29. Devinsky O, Patel AD, Cross H, Villanueva V et al. Effect of Cannabidiol on Drop Seizures in the Lennox-Gastaut Syndrome. N Engl J Med 2018; 378:1888-1897.
30. McCoy B., et al. (2018). “A prospective open-label trial of a CBD/THC cannabis oil in dravet syndrome.” Ann Clin Transl Neurol. 5(9):1077-1088.
31. Johnson, J. R., et al. (2010). "Multicenter, double-blind, randomized, placebo-controlled, parallel-group study of the efficacy, safety, and tolerability of THC:CBD extract and THC extract in patients with intractable cancer-related pain." J Pain Symptom Manage **39**(2): 167-179.
32. Machado Rocha, F. C., et al. (2008). "Therapeutic use of Cannabis sativa on chemotherapy-induced nausea and vomiting among cancer patients: systematic review and meta-analysis." Eur J Cancer Care (Engl) **17**(5): 431-443.
33. Portenoy, R. K., et al. (2012). "Nabiximols for opioid-treated cancer patients with poorly-controlled chronic pain: a randomized, placebo-controlled, graded-dose trial." J Pain **13**(5): 438-449.
34. Tramer, M. R., et al. (2001). "Cannabinoids for control of chemotherapy induced nausea and vomiting: quantitative systematic review." BMJ **323**(7303): 16-21.
35. Kolodny, R. C., et al. (1974). "Depression of plasma testosterone levels after chronic intensive marihuana use." N Engl J Med **290**(16): 872-874.
36. Sarfaraz, S., et al. (2005). "Cannabinoid receptor as a novel target for the treatment of prostate cancer." Cancer Res **65**(5): 1635-1641.

37. Sarfaraz, S., et al. (2006). "Cannabinoid receptor agonist-induced apoptosis of human prostate cancer cells LNCaP proceeds through sustained activation of ERK1/2 leading to G1 cell cycle arrest." J Biol Chem **281**(51): 39480-39491.
38. De Petrocellis, L., et al. (2013). "Non-THC cannabinoids inhibit prostate carcinoma growth in vitro and in vivo: pro-apoptotic effects and underlying mechanisms." Br J Pharmacol **168**(1): 79-102.
39. Turcotte, C., et al. (2016). "The CB2 receptor and its role as a regulator of inflammation." Cell Mol Life Sci **73**(23): 4449-4470.
40. Aaronson NK, Ahmedzai S, Bergman B, Bullinger M, Cull A, Duez NJ, et al. The European Organization for Research and Treatment of Cancer QLQ-C30: A Quality-of-Life Instrument for Use in International Clinical Trials in Oncology. Journal of the National Cancer Institute. 1993;85(5):365-76.
41. Yuan Y, Hess KR, Hilsenbeck SG, Gilbert MR. Bayesian Optimal Interval Design: A Simple and Well-Performing Design for Phase I Oncology Trials. *Clin Cancer Res*. 2016;22(17):4291–4301. doi:10.1158/1078-0432.CCR-16-0592

APPENDIX A: PERFORMANCE STATUS CRITERIA

ECOG Performance Status Scale	
Grade	Description
0	Normal activity. Fully active, able to carry on all pre-disease performance without restriction.
1	Symptoms, but ambulatory. Restricted in physically strenuous activity, but ambulatory and able to carry out work of a light or sedentary nature (*e.g.*, light housework, office work).
2	In bed <50% of the time. Ambulatory and capable of all self-care, but unable to carry out any work activities. Up and about more than 50% of waking hours.
3	In bed >50% of the time. Capable of only limited self-care, confined to bed or chair more than 50% of waking hours.
4	100% bedridden. Completely disabled. Cannot carry on any self-care. Totally confined to bed or chair.
5	Dead.

APPENDIX B. QUALITY OF LIFE:

EORTC's Validated, Patient-Report Questionnaires

Quality of Life: This is assessed by two validated patient-reported outcomes measures, the EORTC QLQ-C30 and QLQ-PR25 questionnaires. All items assessing symptoms and functional domains employ a 4-point Likert scale, with responses ranging from "Not At All", "A Little", "Quite a Bit" and "Very Much." Study participants will be instructed to choose one response per item. The EORTC **QLQ-C30** is a 30-item questionnaire assessing multiple domains of global health-related quality of life in cancer populations. It includes one global QOL rating, 5 functioning scales (physical, cognitive, emotional, role and social) and 9 symptom-specific subscales (fatigue, N/V, pain, dyspnea, insomnia, constipation, diarrhea, appetite loss). The EORTC **QLQ-PR25** is a prostate cancer module meant for use among patients in varying in disease stage and treatment modality (i.e., surgery, chemotherapy, radiotherapy, etc.), which complements the QLQ-C30. The QLQ-PR25 module comprises 25 items assessing disease symptoms, treatment side effects, sexual functioning and sexual activity. The functional scales are sexual activity and sexual functioning; the 4 symptom scales are urinary symptoms, bowel symptoms, hormonal treatment-related symptoms, and incontinence aid. All participants will complete items 31-49 as well as items 50-51 (which query current sexual activity).
For interpretation of scores on the two EORTC QOL questionnaires, all of the scales and single-item measures range in score from 0 – 100. A high scale score represents a higher response level – so that a higher score represents a higher ("better") level of functioning OR a higher ("worse) level of symptoms.

EORTC	Item / Scale	# of Items	Responses	Item No.s	Score Interpretation
QLQ-C30	Global QOL	2	1 - 7	29, 30	Higher score = better global QOL
QLQ-C30 Function Scales	Physical Functioning	5	1 - 4	1-5	Higher score = better functioning
	Cognitive Functioning	2		20, 25	
	Emotional Functioning	4		21-24	
	Role Functioning	2		6, 7	
	Social Functioning	2		26, 27	
QLQ-C30 Symptoms	Fatigue	3	1 - 4	10, 12, 18	Higher score = more/worse symptoms
	Nausea / Vomiting	2		14, 15	
	Pain	2		9, 19	
	Dyspnea	1		8	
	Insomnia	1		11	
	Appetite Loss	1		13	
	Constipation	1		16	
	Diarrhea	1		17	
	Financial Difficulties	1		28	
QLQ-PR25	Sexual Activity	2	1 - 4	50, 51	Higher score = better functioning
	Sexual Functioning *	4		52-55	
QLQ-PR25 Symptoms	Urinary Symptoms	8	1 – 4	31-37, 39	Higher score = more/worse symptoms
	Bowel Symptoms	4		40-43	
	Hormonal Treatment-related Symptoms	6		44-49	
	Incontinence Aid	1		38	

* Completion of Items 52-55 are conditional on being sexually active (and comprise the Sexual Functioning subscale), and thus, these 4 items will only be completed by a subgroup of study patients. Response to item #52 keeps original scoring, while responses to items 53-55 are reverse-scored. Specifically, for items 53-55, a response of "4" is recoded as "1", while a response of "3" is recoded as "2", a response of "2" is recoded as "3", and a response of "1" is recoded as "4"). This reverse-scoring approach preserves the Functional scale property of scoring interpretation, where a higher response indicates higher functioning.

EORTC QLQ-C30 (version 3)

We are interested in some things about you and your health. Please answer all of the questions yourself by circling the number that best applies to you. There are no "right" or "wrong" answers. The information that you provide will remain strictly confidential.

	Not at All	A Little	Quite a Bit	Very Much
1. Do you have any trouble doing strenuous activities, like carrying a heavy shopping bag or a suitcase?	1	2	3	4
2. Do you have any trouble taking a long walk?	1	2	3	4
3. Do you have any trouble taking a short walk outside of the house?	1	2	3	4
4. Do you need to stay in bed or a chair during the day?	1	2	3	4
5. Do you need help with eating, dressing, washing yourself or using the toilet?	1	2	3	4

**During the past week:**	Not at All	A Little	Quite a Bit	Very Much
6. Were you limited in doing either your work or other daily activities?	1	2	3	4
7. Were you limited in pursuing your hobbies or other leisure time activities?	1	2	3	4
8. Were you short of breath?	1	2	3	4
9. Have you had pain?	1	2	3	4
10. Did you need to rest?	1	2	3	4
11. Have you had trouble sleeping?	1	2	3	4
12. Have you felt weak?	1	2	3	4
13. Have you lacked appetite?	1	2	3	4
14. Have you felt nauseated?	1	2	3	4
15. Have you vomited?	1	2	3	4
16. Have you been constipated?	1	2	3	4

Please go on to the next page

**During the past week:**	**Not at All**	**A Little**	**Quite a Bit**	**Very Much**
17. Have you had diarrhea?	1	2	3	4
18. Were you tired?	1	2	3	4
19. Did pain interfere with your daily activities?	1	2	3	4
20. Have you had difficulty in concentrating on things, like reading a newspaper or watching television?	1	2	3	4
21. Did you feel tense?	1	2	3	4
22. Did you worry?	1	2	3	4
23. Did you feel irritable?	1	2	3	4
24. Did you feel depressed?	1	2	3	4
25. Have you had difficulty remembering things?	1	2	3	4
26. Has your physical condition or medical treatment interfered with your family life?	1	2	3	4
27. Has your physical condition or medical treatment interfered with your social activities?	1	2	3	4
28. Has your physical condition or medical treatment caused you financial difficulties?	1	2	3	4

For the following questions please circle the number between 1 and 7 that best applies to you

29. How would you rate your overall health during the past week?

1	2	3	4	5	6	7
Very poor						Excellent

30. How would you rate your overall quality of life during the past week?

1	2	3	4	5	6	7
Very poor						Excellent

EORTC QLQ - PR25

Patients sometimes report that they have the following symptoms or problems. Please indicate the extent to which you have experienced these symptoms or problems during the past week. Please answer by circling the number that best applies to you.

During the past week	Not at all	A little	Quite a bit	Very much
31. Have you had to urinate frequently **during the day**?	1	2	3	4
32. Have you had to urinate frequently **at night**?	1	2	3	4
33. When you felt the urge to pass urine, did you have to hurry to get to the toilet?	1	2	3	4
34. Was it difficult for you to get enough sleep, because you needed to get up frequently at night to urinate?	1	2	3	4
35. Have you had difficulty going out of the house because you needed to be close to a toilet?	1	2	3	4
36. Have you had any unintentional release (leakage) of urine?	1	2	3	4
37. Did you have pain when you urinated?	1	2	3	4
38. Answer this question only if you wear an incontinence aid. Has wearing an incontinence aid been a problem for you?	1	2	3	4
39. Have your daily activities been limited by your urinary problems?	1	2	3	4
40. Have your daily activities been limited by your bowel problems?	1	2	3	4
41. Have you had any unintentional release (leakage) of stools?	1	2	3	4
42. Have you had blood in your stools?	1	2	3	4
43. Did you have a bloated feeling in your abdomen?	1	2	3	4
44. Did you have hot flushes?	1	2	3	4
45. Have you had sore or enlarged nipples or breasts?	1	2	3	4
46. Have you had swelling in your legs or ankles?	1	2	3	4

Please go to the next page

**During the last 4 weeks...**	**Not at all**	**A little**	**Quite a bit**	**Very much**
47. Has weight loss been a problem for you?	1	2	3	4
48. Has weight gain been a problem for you?	1	2	3	4
49. Have you felt less masculine as a result of your illness or treatment?	1	2	3	4
50. To what extent were you interested in sex?	1	2	3	4
51. To what extent were you sexually active (with or without intercourse)?	1	2	3	4

PLEASE ANSWER THE NEXT FOUR QUESTIONS ONLY IF YOU HAVE BEEN SEXUALLY ACTIVE OVER THE LAST 4 WEEKS

52. To what extent was sex enjoyable for you?	1	2	3	4
53. Did you have difficulty getting or maintaining an erection?	1	2	3	4
54. Did you have ejaculation problems (eg dry ejaculation)?	1	2	3	4
55. Have you felt uncomfortable about being sexually intimate?	1	2	3	4

APPENDIX C: PATIENT DRUG INTERACTIONS

An example list of drugs that can have major/moderate interactions with Epidiolex or CBD. For a list of drugs that have minor interactions with Epidiolex, please refer to Micromedex or other frequently updated lists.

DRUG CLASS	DRUG NAME
Patients are **ineligible** for GU-74 trial if taking either of these two meds (per Section 3.2.7)	Clobazam (Onfi, Sympazan): patients are ineligible for this trial<br>Valproate (Depakote): patients are ineligible for this trial
CYP2C19 sensitive substrates are allowed to be used with caution and consideration of reduction of Epidiolex dose (Section 6.5):	Carisoprodol<br>Citalopram<br>Clopidogrel<br>Diazepam<br>Lansoprazole<br>Omeprazole<br>Phenytoin<br>Tofacitinib
Medications affecting CYP2C19 (moderate/potent inhibitors or inducers) are allowed to be used with caution and consideration of reduction of Epidiolex dose (Section 6.5):	Warfarin<br>Proton pump inhibitors (PPIs): Esomeprozole, Pantoprazole<br>Anti-epileptics: S-mephenytoin, Phenobarbitone, Oxcarbazepine<br>Anti-microbials: Isoniazid, Voriconazole<br>Anti-depressants: Amitriptyline
Medications affecting CYP3A4 are allowed to be used with caution and consideration of Epidiolex dose reduction (Section 6.5):	Amiodarone Clarithromycin Cyclosporine Ritonavir<br>Conviaptan Aprepitant Amprenavir Verapamil<br>Diltizem Erythromycin Voriconazole<br>Telithromycin Saquinavir Darunavir<br>Posaconazole Curcumin Midostaurin<br>Telaprevir Idealisibl Stripentol<br>Itraconazole Nelfinavir Ketoconazole
Medications affecting CYP2D6 are allowed to be used with caution with CBD:	Amiodarone (Cordarone)<br>Bupropion (Wellbutrin)Chlorpheniramine (Chlor-Trimeton)<br>Chloroquine (Aralen)<br>Chlorpromazine (Thorazine)<br>Cinacalcet (Sensipar)<br>Diphenhydramine (Benadryl)<br>Duloxetine (Cymbalta)<br>Fluoxetine (Prozac)<br>Halofantrine (Halfan)<br>Haloperidol (Haldol)<br>Imatinib (Gleevec)<br>Paroxetine (Paxil)<br>Perphenazine (Trilafon)<br>Propafenone (Rythmol)<br>Propoxyphene (Darvon)<br>Quinacrine (Atabrine)<br>Quinidine (Quinidex, etc)<br>Quinine<br>Terbinafine (Lamisil)

APPENDIX D: DRUG ACCOUNTABILITY FORM

Markey Cancer Center Patient Self-Administered Study Agent Compliance Log

This form is to be updated at every study contact where patient receives or returns study drug. This form may be used for multiple self-administered study agents. This form is to be used in conjunction with a note in SCM about study drug self-administration and case report form and will be maintained in the research record.

MCC Protocol Number:__MCC-19-GU-74_____ Principal Investigator: ___Zin Myint, MD___________________

Protocol Title: __Epidiolex in Patients with Rising PSA____

Patient's Medical Record Number:____________________ Patient's Name:_________________________________

Patient Study ID: ________________Cycle Number : ______________________________

Date Dispensed	Amount Dispensed	Dose Form *(e.g., tablets, pills, bottles, capsules, syringes, vials)*	Date Returned	Actual Amount Returned	Expected Amount Taken	Expected Amount Returned	Reason for difference between actual and expected amount returned, if applicable	Site Staff Initials
____/_____/20_____ (mm/dd/yyyy)			____/_____/20______ (mm/dd/yyyy)					
____/_____/20_____ (mm/dd/yyyy)			____/_____/20______ (mm/dd/yyyy)					
____/_____/20_____ (mm/dd/yyyy)			____/_____/20______ (mm/dd/yyyy)					
____/_____/20_____ (mm/dd/yyyy)			____/_____/20______ (mm/dd/yyyy)					
____/_____/20_____ (mm/dd/yyyy)			____/_____/20______ (mm/dd/yyyy)					
____/_____/20_____ (mm/dd/yyyy)			____/_____/20______ (mm/dd/yyyy)					
____/_____/20_____ (mm/dd/yyyy)			____/_____/20____ (mm/dd/yyyy)					

Version 1, 28Aug2019- SOP Documenting Return of Oral Agents Self-administered by Patients Page ____ of ____

Markey Cancer Center
Investigational Drug Return Form

Patient Number: ____

Protocol Number: ____

Name of Investigational Drug: _________________________________

Principal Investigator: ____

Email address of MCC CRO/PMC personnel(s) to be notified of discrepancies in final total Investigational Drug returned count:

____

____

____

Date of Investigational Drug Drop off: ______________________________

Total amount of drug returned to IDS Pharmacy (# capsules/tablets) ___________

Name of MCC CRO/PMC personnel delivering returned Investigational Drug:
____

CRA/CRN Signature: ______________________ Date: ___________

APPENDIX E: PATIENT INSTRUCTIONAL HANDOUT FOR EPIDIOLEX

Epidiolex (cannabidiol)

How to Take EPIDIOLEX

Follow the steps below to learn how EPIDIOLEX is prepared, taken, and readied for the next dose. If you have any questions, ask your doctor or specialty pharmacy.

Putting on the Bottle Adapter

1. Remove bottle cap.
2. Push the bottle adapter firmly into the bottle. Do not remove the adapter after it has been inserted.

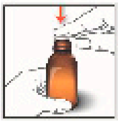

Tip: If you have trouble getting the bottle adapter on, insert the syringe into the bottle adapter and use that to help fit it onto the bottle.

How to Prepare the Right Dose

1. Push the plunger all the way down and insert the tip of the syringe into the bottle adapter. Turn the bottle upside down.
2. Slowly pull the plunger to withdraw the dose.

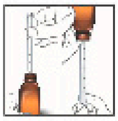

3. Line up the end of the plunger with the marking for your dose.

   If you see air bubbles, flick the syringe to get the air bubble to the top then push it back into the bottle. Repeat the process until the air bubbles are gone.

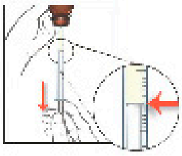

4. Leave the syringe in and turn the bottle right side up.
5. Carefully remove the syringe.

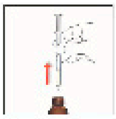

How to Give EPIDIOLEX

1. Place the tip of the syringe against the inside of the cheek and gently push the plunger until all the EPIDIOLEX is given.

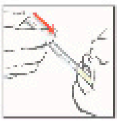

- **Do not** force the plunger down.
- **Do not** direct the medicine to the back of the mouth or throat. This may cause choking.

Dosing Tips

If the prescribed dose is more than 5 mL, you'll need to fill the syringe twice. For example, for an 8 mL dose, first withdraw 5 mL of EPIDIOLEX, administer it, and then withdraw another 3 mL.

If the prescribed dose is not a whole number, let's say 3.8 mL, here's what you do: Draw up to the 3 mL line and then continue drawing up past the 3.5 mL line until you reach 3.8 mL.

If you were sent 1 mL syringes, you can prepare a dose as described in this section. For example, if the dose is .35 mL, you will draw up to the .3 mL line, and then go 1 hashmark farther to .35 mL. If the prescribed dose is between hashmarks, ask your doctor for instructions on rounding up or down to the nearest marking.

SELECT IMPORTANT SAFETY INFORMATION

EPIDIOLEX may cause liver problems. Your doctor may order blood tests to check your liver before you start taking EPIDIOLEX and during treatment. In some cases, EPIDIOLEX treatment may need to be stopped. Call your doctor right away if you start to have any of these signs and symptoms of liver problems during treatment with EPIDIOLEX:

- loss of appetite, nausea, vomiting
- fever, feeling unwell, unusual tiredness
- yellowing of the skin or the whites of the eyes (jaundice)
- itching
- unusual darkening of the urine
- right upper stomach area pain or discomfort

Please see full Important Safety Information on the last page.
Please refer to the Prescribing Information, including the Medication Guide and Instructions for Use on EPIDIOLEX.com.

APPENDIX F: PATIENT MEDICATION DIARY

Two patient diary (medication logs) were created –

1. A 30-day diary for Cycles 1 – 3 (e.g., Days 1 - 90). This diary is two-pages with Days 1 – 30 noted, and a few empty lines for entry to use as needed (e.g., if the next clinic visit is a few days beyond the 30-days of the cycle, such as falling on a weekend or holiday).

2. For Cycle 3 patients receive the 30-day log PLUS an additional, separate page for recording compliance with the 10-day taper of Epidiolex.

30-Day Diary of Epidiolex

Day	Date	Dose	Time	Mark an "X" in this column if dose was missed.	Side Effects
Example of a completed log for three days (Day 15 – Day 17).					
**15**	*7/1/20*	*600mg*	*8 PM*		*None.*
**16**	*7/2/20*	*----*	*-----*	*X*	*Missed the dose today.*
**17**	*7/3/20*	*600mg*	*8:15 PM*		*Nausea, mild – went away after an hour.*
	**Date**	**Dose**	**Time**	**Mark "X" in this column <u>if</u> you missed the dose.**	**Side effects**
**1**					
**2**					
**3**					
**4**					
**5**					
**6**					
**7**					
**8**					
**9**					
**10**					
**11**					
**12**					
**13**					
**14**					
**15**					
**16**					

**17**					
**18**					
**19**					
**20**					
**21**					
**22**					
**23**					
**24**					
**25**					
**26**					
**27**					
**28**					
**29**					
**30**					

Daily Log of Epidiolex During the 10-day Taper

Your daily dose will be *slowly reduced* over a 10-day period.
Your doctor will write in the correct dose that you are to take for each day.
Please call your cancer doctor if you have any questions about this.

Day	Date	Dose	Time	Mark an "X" in this column if this dose was missed.	Side Effects
1					
2					
3					
4					
5					
6					
7					
8					
9					
10					
